# Exploring polymorphisms in B-DNA helical conformations

**DOI:** 10.1093/nar/gks884

**Published:** 2012-09-24

**Authors:** Pablo D. Dans, Alberto Pérez, Ignacio Faustino, Richard Lavery, Modesto Orozco

**Affiliations:** ^1^Joint IRB-BSC Program on Computational Biology, Institute for Research in Biomedicine (IRB), Parc Cientific de Barcelona, Josep Samitier 1-5, Barcelona 08028, Spain, ^2^Institut Pasteur de Montevideo, Mataojo 2020, Montevideo 11400, Uruguay, ^3^Laufer Center for Physical and Quantitative Biology, Stony Brook University, Stony Brook, NY 11794-5252, USA, ^4^Barcelona Supercomputing Centre (BSC), Jordi Girona 31, Edifici Torre Girona, Barcelona 08034, Spain, ^5^Bioinformatics: Structures and Interactions, Bases Moléculaires et Structurales des Systèmes Infectieux, Univ. Lyon I/CNRS UMR 5086, IBCP, 7 Passage du Vercors, Lyon 69367, France and ^6^Departament de Bioquimica, Facultat de Biologia, Avgda Diagonal 647, Barcelona 08028, Spain

## Abstract

The traditional mesoscopic paradigm represents DNA as a series of base-pair steps whose energy response to equilibrium perturbations is elastic, with harmonic oscillations (defining local stiffness) around a single equilibrium conformation. In addition, base sequence effects are often analysed as a succession of independent XpY base-pair steps (i.e. a nearest-neighbour (NN) model with only 10 unique cases). Unfortunately, recent massive simulations carried out by the ABC consortium suggest that the real picture of DNA flexibility may be much more complex. The paradigm of DNA flexibility therefore needs to be revisited. In this article, we explore in detail one of the most obvious violations of the elastic NN model of flexibility: the bimodal distributions of some helical parameters. We perform here an in-depth statistical analysis of a very large set of MD trajectories and also of experimental structures, which lead to very solid evidence of bimodality. We then suggest ways to improve mesoscopic models to account for this deviation from the elastic regime.

## INTRODUCTION

DNA is a large and flexible polymer that can easily change its conformation to adapt to different interactions (1). Early models of flexibility assumed averaged sequence-independent elastic properties for DNA (2–4). Despite this extreme simplicity, these models were successful in describing several macroscopic properties of long fragments of DNA (4,5). However, it very soon became evident that not all DNA sequences respond in the same way to mechanical stress, implying that sequence-dependent models of DNA flexibility needed to be developed (6–10). Since their origins, such models have become very valuable for investigating the connection between the sequence-dependent physical and biological properties of DNA (11–13), shedding light on such important processes as the mechanisms of chromatin organization, or the physical basis of the indirect recognition of DNA by regulatory proteins. The importance of these models relies on their utility to link microscopic observables with macroscopic properties, and to bridge the gap between the size of the systems and the length of simulations that could be achieved with atomistic approaches and the so-called ‘mesoscale’ where many of the most important biological events take place.

The most popular sequence-dependent flexibility models of DNA assume that, first, DNA responds elastically to mechanical stress, and, second, that sequence effects are fully characterized at the base-pair step level (6,9,14). This implies that there is a quadratic dependence between the degree of geometrical deformation and the distortion energy, and also that the parameters defining such a response (namely the equilibrium value and the force-constant) can be obtained using the nearest-neighbours (NNs) approach, that is, for a given helical descriptor of DNA there are only 10 sets of different parameters (those corresponding to the 10 unique dinucleotide steps: d(AA)·d(TT), d(AG)·d(CT), d(AC)·d(GT), d(AT)·d(AT), d(GG)·d(CC), d(GA)·d(TC), d(GC)·d(GC), d(TA)·d(TA), d(TG)·d(CA) and d(CG)·d(CG). The most popular mesoscopic model of flexibility originated from the Zhurkin and Olson groups (6) and describes flexibility in terms of equilibrium values and stiffness parameters associated with six inter-base-pair coordinates (twist, roll, tilt, slide, shift and rise). Olson *et al.* (6) derived the parameters of their model by making Gaussian fits to the helical parameter distributions for each type of dinucleotide step within a database of DNA–protein complexes. Then, maximum probability peaks are equated to equilibrium values and the widths of the distributions to the associated stiffness constants (corresponding to the diagonal elements of the full stiffness matrix). Lankas *et al.* (15) refined Olson’s model with the help of conformational sampling from molecular dynamics (MD) trajectories of short duplexes (9,16), which allowed them to obtain dense and homogeneous data for all steps in naked DNA and enabled both diagonal and off-diagonal elements of the stiffness to be derived. However, both these models assumed the NN description of sequence effects and simple elastic deformations.

In a massive community effort for characterizing B-DNA flexibility, a large number of oligonucleotides containing the 136 unique tetranucleotide steps were studied by means of atomistic MD simulations (17). The results provided a detailed and balanced map of DNA flexibility, but also unexpected and intriguing features: (i) in many cases non-neighbour effects modify the conformational preferences of the dinucleotide steps; and (ii) some non-normal distributions were detected. Notably, bimodal distributions were observed in some base-pair steps for twist and slide (17), highlighting potential caveats of the NN model and also of the harmonic approximation implicit in elastic analysis (18). It however remains to be shown that the bimodality detected in ABC simulations is not a force-field artefact, or an equilibration issue related to the length of trajectories. If verified, it is also unclear how these effects should be accounted for in mesoscopic models of DNA flexibility.

We report here the results of a very large-scale analysis of MD trajectories from our local trajectory database, some of them covering multi-microsecond ensembles, in addition to the ABC dataset and also experimental DNA structures deposited in the Protein Data Bank (PDB). This conformational data were processed by using a wide repertory of statistical tests, including the Bayesian Information Criterion (BIC; (19)), Bayes Factors metrics (20) and generalized Helguerro’s theory (21). The results, while raising concerns for some of the conclusions derived from ABC data, provide solid support for others, suggesting that deviation from the NN-elastic flexibility paradigm can no longer be neglected.

## MATERIALS AND METHODS

The experimental conformational space, defined as a set of experimental structures in the PDB, was compared with a collection of structures taken from many independent MD trajectories (considered as a single theoretical conformational space by combining all the trajectories). The aggregated simulation time (for 160 individual trajectories) presented here corresponds to more than 10 µs of simulation, and nearly reaches 1 ms of sampling for some base-pair steps.

### Structural database

The 974 DNA segments analysed in this work were obtained from 739 different PDB files determined experimentally by X-ray techniques. Structure files were collected from three on-line databases. (i) The 3DNA Landscapes database (http://3dnascapes.rutgers.edu/) (22); (ii) the Protein–DNA Interface database implemented by Norambuena and Melo (http://melolab.org/pdidb/web/content/home) (23); and finally (iii) the Nucleic Acid Database (NDB; http://ndbserver.rutgers.edu/) (24). On-line filters provided by the databases were applied and in-house post-processing was performed to ensure data quality. Thus, only X-ray structures with resolution 2.5 Å or better were selected. Sequences with three or more modified bases, and those containing non-canonical covalent bonds (e.g. thymine dimers, covalently bound DNA–protein or DNA–DNA complexes) were removed. When downloading naked-DNA structures, we only retain DNA segments in the B-form (more than 50% of the base pairs in the B-form, according to the criterion used in the 3DNALandscapes database (22) based on the positions of P atoms within individual base-pair steps). Finally, base-pair steps with anomalous helical parameters (more than three standard deviations from the mean of the measured properties) were not included in the statistical tests. Analysis was always limited to canonical bases in their major tautomeric and ionic states.

After the selection procedure 7685 base-pair steps remained: 86% come from DNA–protein complexes and 14% correspond to naked-DNA structures (i.e. structures without protein or ligands in the crystal). The set of DNA–protein structures is composed of 58% of enzymes, 31% of transcription factors and 10% of structural binding proteins. To explore the potential impact of non-normality in modulating DNA interactions we also created an additional dataset using the X-Ray structures of complexes of DNA with intercalators. The corresponding PDB entries of all the files organized in these categories are available in the Supplementary Data (Table S1).

### MD dataset

We analysed the flexibility of the different base-pair steps in our local database of trajectories, which contains trajectories typically in the 50–200 ns range, plus all the trajectories of the different tetramers retrieved from the ABC consortium (17). All trajectories were obtained using state-of-the-art simulation conditions (16,17,25) and the parmbsc0 refinement (26) to the parm99 force field (27). Simulations were done using either SPC/E (28) or TIP3P (29), water models (we did not find any major difference between results obtained with these two solvent models), K^+^ or Na^+^ were used as counterions, some calculations were done with minimum cation concentrations to achieve neutrality, others were performed using small quantities of added salt (KCl or NaCl), and in a few cases we used higher ionic strength (again, our analysis did not show any significant dependence of DNA properties on salt concentration, at least up to 500 mM added salt).

The resulting dataset of trajectories covers the entire sequence space (up to the tetramer level) and represents more than 10 µs for the ensemble (Supplementary Table S2), and nearly a millisecond of aggregated sampling for some steps. In addition, in order to analyse convergence issues, we extended our Drew–Dickerson dodecamer simulation (25) to 4 µs, which additionally allows us to determine the expected timescale of multimodal transitions in B-DNA. To achieve the statistical test described below, 1 million structures were randomly extracted from the ensemble generated for each bps. This procedure was repeated more than once to ensure that the statistical measures did not change with the sampling methods.

### Structural analysis of the data

All the experimental structures were visually inspected using VMD 1.9 (30) and hand-curated when necessary. Before making any measurements, all non-DNA molecules were removed. Six inter-base pair helical parameters were measured (rise, roll, shift, slide, tilt and twist) using the Curves+ suite of programs (31).

### Statistical analysis of data

A normal distribution is a continuous probability distribution used to describe real-valued variables that tend to cluster around a single average value. This distribution follows a well-known Gaussian function:
(1)
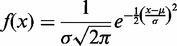

where, *σ* is the standard deviation of the variable *x* and *μ* the average value of the distribution. Note that a normal distribution implies a harmonic response of the variable *x* to deviations from the mean value *μ*. For molecular systems, a Gaussian distribution for a given coordinate implies a harmonic response to perturbations of the geometry with respect to such a coordinate:
(2)
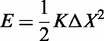

where *K* is the stiffness constant associated to the variable and Δ*X = x −X_0_* is the deviation of the variable from the equilibrium value (*X_0_*).

A normal distribution is always symmetrical and unimodal (in this particular case the average and the mode, i.e. the most frequent value in the sample, have exactly the same value). Deviations from normality can be of many different types. One very common deviation is asymmetry (skewness) of the distribution about the mean. Another common deviation is multimodality (particularly bimodality), where instead of a continuum distribution centred on one mode there is a continuum distribution centred on two, or more, different local means (32). In some cases, bimodal distributions can be fitted to binormal distributions, which are defined as a combination of two Gaussian functions (21):
(3)
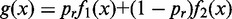

where *f*_1_(*x*) and *f*_2_(*x*) are the two Gaussian components of the binormal distribution *g*(*x*), and *p_r_* is the mixture proportion.

#### Bayesian information criterion

This criterion serves for model selection, i.e. find a model that allows to describe the distribution of our data using a finite set of known functions (in this case, Gaussian functions) (19). BIC is used here to test for the presence of uninormality, binormality or multinormality depending on the number of Gaussian functions used to fit the data. This approach will optimally fit a given distribution with an (*a priori*) undefined number of parameters, i.e. an undefined number of Gaussian, by finding the set of parameters that minimizes the BIC (the model with the lower BIC is then chosen) (19):
(4)
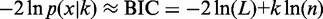

where *x* are the observed data, *k* is the number of free parameters to be estimated, and *p*(*x|k*) is the probability of the observed data given the number of parameters, or, in other words, the likelihood of the parameters given the dataset. *L* is the maximized value of the likelihood function for the estimated model, and *n* is the number of data points in *x* (the number of observations, or equivalently, the sample size). Note that BIC controls the introduction of extra-fitted parameters (second term in equation (4)), which will be accepted only when they significantly improve the representation of the distribution. To reduce the risk of over-fitting (given that some distributions are relatively sparse) we limit BIC to considering a maximum of two Gaussians.

#### Bayes factor analysis

This method was used to process the BIC values obtained in the statistical analysis described above. It allows determining the strength of the evidence in favour of the model chosen by BIC (uninormal or binormal). The difference in BIC between two models *i* and *j* is ≈ −2 times the natural logarithm of the Bayes factors (*F*) for model *j* versus model *i* (20):
(5)
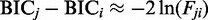

and thus, to a good approximation, the Bayes factors are related to the BIC values as follow:
(6)
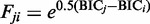

Assuming that there is an equal probability of obtaining an explanatory model with one (*M*1) or with two components (*M*2), with BIC values BIC_1_ for *M*1 and BIC_2_ for *M*2, the probability of a two-component model being justified is defined as (20):
(7)
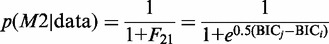

Considering a pre-determined confidence level of 5%, we consider that there is strong evidence in favour of *M*2 (i.e. the need for combining two normal distributions) if *p*(*M*2*|*data) > 0.95 and strong evidence in favour of *M*1 if *p*(*M*2|data) < 0.05. Between these two extreme values, we should conclude that there is insufficient evidence (IE) to support either models. Considering this range of possibilities, results are reported as *M*1 (uninormal model supported), *M*2 (binormal model supported), or IE.

#### Testing modality

Despite the power of the BIC in distinguishing between uni- and binormality, the concept of modality was introduced as an additional criterion to distinguish binormal distributions where the two peaks (the two modes) are close together from those where they are significantly separated. This is the most important distinction in terms of understanding DNA dynamics. In the first case, for practical purposes, the use of a single normal distribution may often be justified to represent the data (the overall distribution may be interpreted as unimodal), while it cannot be in the second (binormal-bimodal distributions). The modality for the mixture of two Gaussian functions can be measured quantitatively through the generalization of Helguerro’s theorem (21,33), which defines the separation factor *S*(*r*)*,* based on the variance σ^2^, as
(8)
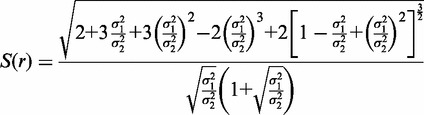

Then the mixture density *g*(*x*) is unimodal regardless of the mixture proportion *p_r_* if and only if:
(9)
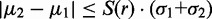

and, otherwise, is bimodal (21). From here on, when *g*(*x*) indicated unimodal or bimodal distributions, they are labelled U or B, respectively.

## RESULTS AND DISCUSSION

### MD versus X-ray structures: equilibrium helical parameters

Table S3 presents the average values and standard deviations of the inter-base pair parameters (note that here, as a first step, we assumed normality as in previous studies with smaller databases (6,9,14,16)). We found that there are cases where the equilibrium values obtained from the analysis of naked-DNA structures are different to those obtained from the analysis of DNA–protein complexes. Some of these differences (Table S3) are so large that they cannot be fully explained just by considering the scarcity of structures in the naked-DNA database, suggesting that either binding to the protein induces non-negligible changes in the DNA conformation, or alternatively that packing effects are significant in small, naked-DNA oligomers. Comparing simulation data with experimental structures for naked DNA, we found that for 85% of the 60 cases studied (6 parameters × 10 bp) the agreement between simulations and X-ray structures is very good. In most of the cases of significant differences between experiment and theory, the experimental data are so scarce that the corresponding averages are not robust (this is the case, e.g., for AC, AG and TA steps). In the cases where there is enough experimental data to obtain robust averages, differences between simulation and X-ray structures in PDB can be justified either by the lack of convergence and/or force-field inaccuracies on the MD-side, or to protein-induced distortions and/or crystal packing artefacts on the X-ray side (for the rotational parameters the differences can be as large as 10°, and in turn more than 1 Å in the translational parameters). Overall, we should stress the ability of simple force-field calculations to reproduce known experimental information on DNA, at least at the base-pair level.

### Helical distributions from MD ensembles and X-ray structures

**Figure 1.**
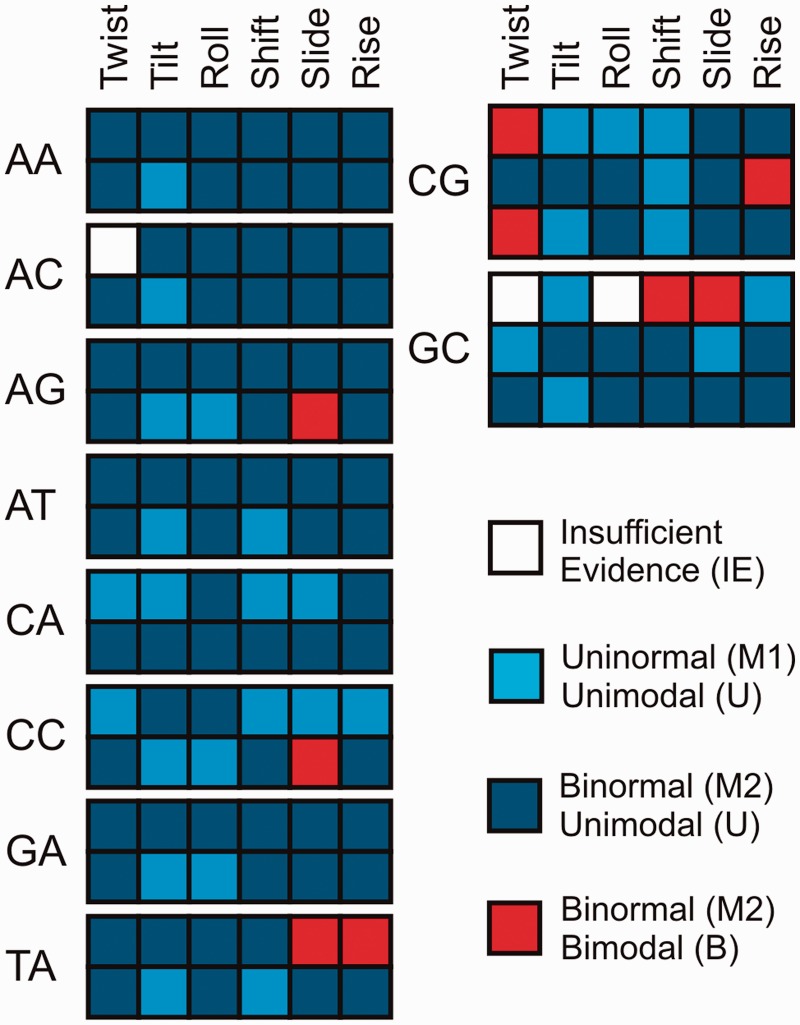
Normality and modality of the 6 inter-base pair helical parameters for the 10 unique base-pair steps. Within each bp (indicated on the left), the complete conformational space of X-ray structures (first row) is compared with the complete MD simulations space (second row). For CG and GC (for which we have enough experimental data on naked DNAs) the comparison (on the right) is made between: naked-DNA structures (first row), all the X-ray structures (second row) and MD simulations (third row).

A careful statistical analysis (see Materials and Methods section) of the experimental and MD-derived datasets reveals that 76% of the distributions fit better to binormal rather than to normal distributions (Figure 1). There are even some cases where more than two normal distributions should be considered for an ideal fit. However, even if most distributions are binormal (*M*2), the individual Gaussians determined by Bayesian analysis (BIC) in many cases have significant overlaps (Supplementary Table S4). Following Helguerro’s theorem this means that most of these distributions can nevertheless be considered as unimodal (U). In these cases (M2/U, dark blue squares on Figure 1), the distributions can be characterized by a single set of weighted averages and standard deviations (Supplementary Table S4), and elastic models are still valid.

We will analyse in more detail in the following the cases for which binormality and bimodality seem supported, since from a structural point of view the finding of binormal-bimodal cases indicate the presence of polymorphism, i.e. the existence of different possible conformations for certain helical parameters where nucleotides are in two or more, discrete (possibly, partially overlapping) conformations.

Bimodal distributions are found for slide and rise in the X-ray DNA–protein distributions, and are present for slide and twist in the MD database. They also appear for shift, slide and twist in the X-ray naked-DNA database (even though here the analysis is limited by the scarcity of data for most steps). Analysis of the dataset reveals that the rise bimodality found in the X-ray structures for CG and TA steps is the result of large, anharmonic perturbations induced by the proteins, namely the partial or complete intercalation of amino acid side chains in these steps (e.g. see the PDB entries: 1CDW, 1D3U, 1BF4 and 1EWQ). In several cases, the bimodality found in slide for TA steps is directly correlated with the bimodality observed in rise (data not shown). No indication of bimodality is found in these cases in the naked-DNA structures, or in the MD sampling. Accordingly, in these cases, we can conclude that proteins distort the DNA away from the elastic regime valid for a naked-DNA structure. In contrast, for the unperturbed DNA, there is no spontaneous sampling of two distinct conformational regions and its behaviour remains unimodal. Bimodal distributions were however found for the shift and slide of GC steps in the X-ray naked-DNA database. These distributions are wide for shift and asymmetric and weighted towards lower values for slide, although again the statistical results are limited by the scarcity of data (for the same number of occurrences, the Bayes factors were unable to support the normality for twist and roll). No matching behaviour was found with the protein–DNA structures, or with the MD ensemble, for either shift or slide, raising doubts as to whether these bimodality signals can be fully justified.

**Figure 2.**
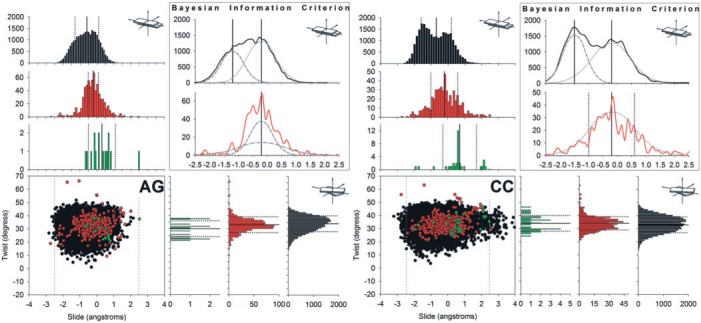
Scatter plots in the slide-twist plane of the AG, and CC base-pair steps (left, and right, respectively), for MD simulations (black), all PDB files (red) and naked-DNA structures (green). In the scatter plot, the grey vertical-dashed lines define the range of values used for twist in the BIC analysis. Histograms on the edges of the scatter plot represent the non-normalized distributions (count). The upper right quadrant shows the results of the analysis carried out with BIC for twist. The Gaussian curves in dashed grey are a qualitative representation of the normal components obtained, whereas the vertical lines represent the corresponding averages (solid) and standard deviations (dashed). For sake of clarity, only a subset of 25000 conformations from the MD simulations was used to build the scatter plot and corresponding histograms.

**Figure 3.**
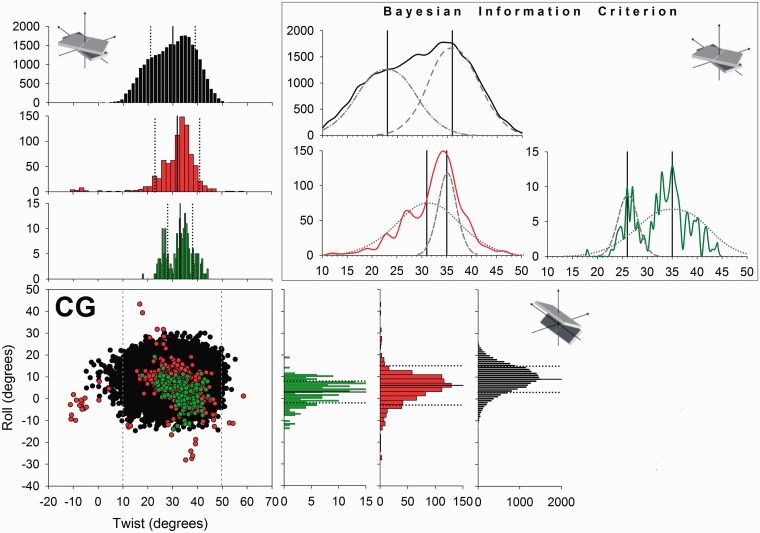
Scatter plot in the twist-roll plane of the CG base-pair step for MD simulations (black), all PDB files (red) and naked-DNA structures (green). In the scatter plot, the grey vertical-dashed lines define the range of values used for twist in the BIC analysis. Histograms on the edges of the scatter plot represent the non-normalized distributions (count). The upper right quadrant shows the results of the analysis carried out with BIC for twist. The Gaussian curves in dashed grey are a qualitative representation of the normal components obtained, while the vertical lines represent the corresponding averages. For sake of clarity, only a subset of 25000 conformations from the MD simulations was used to build the scatter plot and corresponding histograms.

A detailed analysis of the different helical distributions is time consuming, but also extremely informative (see, e.g. Figures 2–3 and Supplementary Figures S1–S4). Among other observations, we find the well-known coupling (6) of some helical deformations (e.g. the negative correlation of twist and roll), and these trends are evident in all the datasets. As found in previous studies (14), MD reproduces the shape of experimental distributions well and, interestingly, MD-derived sampling easily covers the range of DNA conformations seen not only in naked DNA, but also in DNA–protein crystals. Considering that the aggregated MD data considered here spans the micro to millisecond timescale, this implies that most DNA–protein binding, at least locally (at the base-pair step level), could obey the ‘conformational selection’ paradigm (34), since the thermally induced oscillations of naked DNA encompasses the distortions required for protein binding. However, analysis of the crystal structures lying outside the MD conformational distributions also reveals clear, but infrequent, cases of induced fit mechanisms (35) involving large, anharmonic deformations of DNA (see Discussion on unusual rise above and examples in Supplementary Figures S1–S4), well beyond the grasp of elastic models. These results are only part of the complete evidence needed to conclude on the relative importance of ‘conformational selection’ versus ‘induced fit’ paradigms. This would require computing the correlated conformational changes in many parameters, over many base-pair steps, something that is far beyond the present study.

MD simulations of naked DNA found evidence of bimodality in slide for AG and CC, and also in twist for CG. A detailed analysis of the slide distributions in AG and CC (Figures 1 and 2) reveals, that in both these cases, bimodality is related to the population of a secondary free energy minima located at negative slide values (around − 1.5 Å). These minima are populated in protein–DNA complexes (Figure 2) and, in fact, some qualitative similarity exists between MD and protein–DNA X-Ray distributions (Figure 2), although the statistical tests failed to report bimodality for the corresponding DNA–protein distributions. This, combined with the reduced number of naked DNA complexes in experimental database, precludes a direct experimental validation of the bimodality of the slide distributions for AG and CC steps. There is thus currently insufficient experimental data for ApG and CpC slide to support the two-state polymorphism observed in MD simulations.

**Figure 4.**
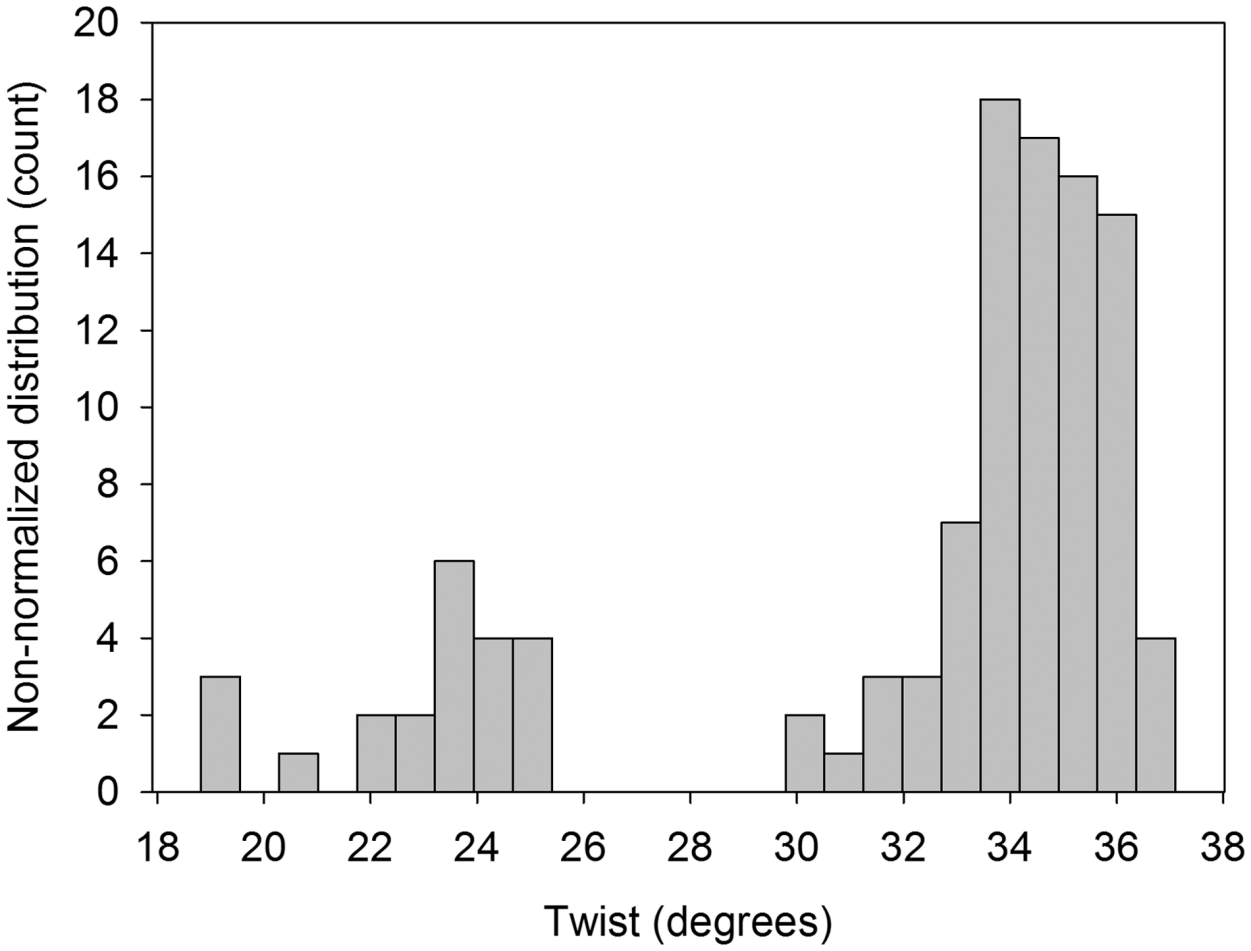
Twist distribution for CG dinucleotide steps found in X-ray structures of non-covalent complexes of DNA with intercalators. Only the CG bps interacting with the intercalators are shown.

Bimodality in twist at CG steps in the MD trajectories corresponds to populating a second minimum in a region of low twist (around 25°; Figure 3). In this particular case, the naked-DNA database is sufficiently populated (more than 200 occurrences) to allow us to use Bayesian statistics and to confirm the presence of polymorphism in the CG twist of the experimental DNA structures. The analysis of DNA–protein structures also reveals binormality, since a simple visual inspection (Figure 3) suggests the presence of a secondary peak of probability at low twist. However, the two fitted distributions (for low and high twist) show significant overlap and Helguerro’s metric reveals that use of a bimodal distribution is not warranted. It therefore seems that proteins tend to favour high-twist CG conformations. Bimodality is however very clear (Figure 4) if we consider DNA duplexes bound to intercalators. These molecules, which have a very marked preference for CG steps, often lead to untwisting DNA at the intercalation site, with twist values typically in the range 22°–25°, corresponding to the low-twist values expected for the minor peak of the bimodal distribution found in MD ensembles of naked DNA. Clearly, spontaneous bimodality appears to be a major factor in explaining the sequence preference of a number of intercalators.

### Origin of bimodality: intrinsic bimodality versus non-NN effects

**Figure 5.**
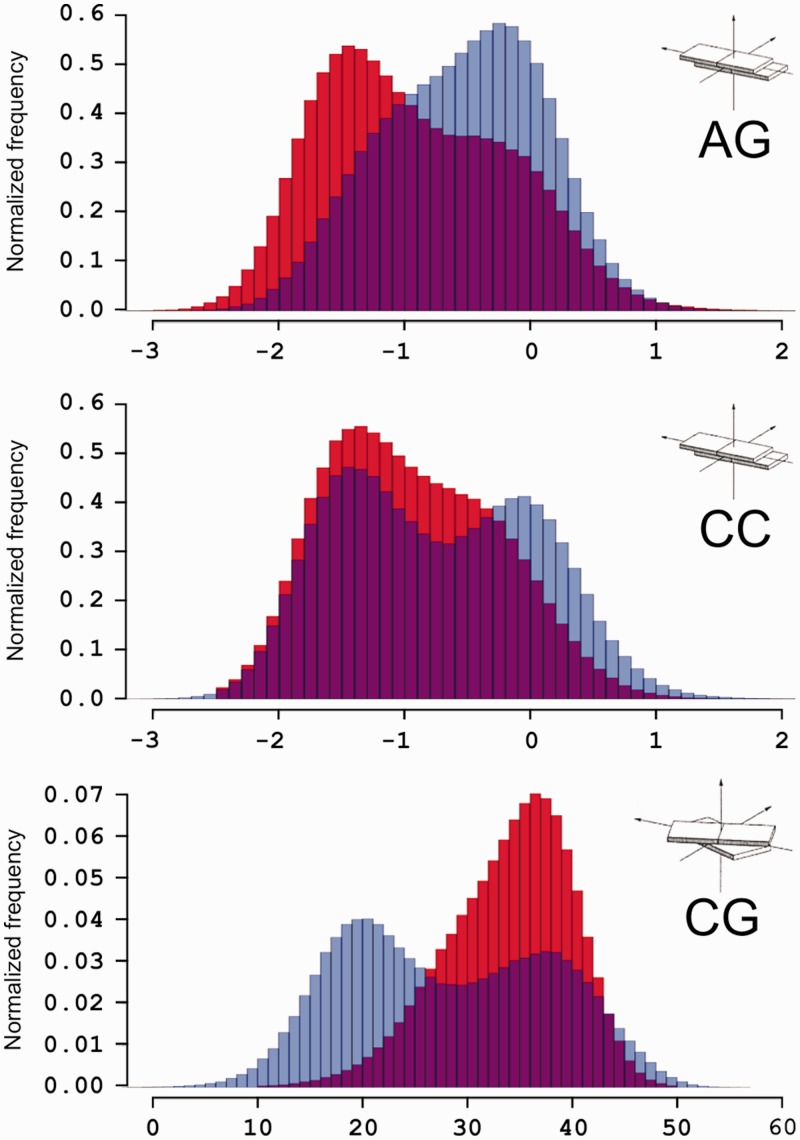
Comparison of the normalized frequency distributions obtained from the original ABC simulations (blue bars) and the four sequences extended in this work (red bars). Three bps are shown: AG and CC for slide, CG for twist (from top to bottom). The overlap between the two histograms is shown in purple.

Bimodality, as described up to this point, can occur in two different situations: (i) two different equilibrium geometries are sampled depending on next-nearest neighbouring nucleotides (i.e. equilibrium parameters for AB in XABY and ZABW environments are significantly different); and (ii) bimodality is intrinsic for a given step and two distributions are visible within a single tetramer environment. The first case will challenge the NN model, but not the elastic hypothesis, since the harmonic approach could still be used by using tetramer rather than dimer-linked parameters (17). The second case will be much more challenging, since it would imply a direct challenge to the elastic response model. ABC trajectories (17) contain at least three independent ensembles for each tetramer within the set of 136 unique cases. This allows us to explore whether bimodality is an intrinsic property, or just a consequence of the use of the NN model. Results shown in Supplementary Figure S5 clearly suggest that bimodality can be an intrinsic property of some DNA steps, since two distributions are visible within a number of tetranucleotide environments. However, caution should be taken with these ABC results, since 50 ns sampling might not allow full convergence of trajectories. To support then our suggestions of bimodality, we extended four ABC sequences up to 750 ns. Results displayed in Figure 5 and Supplementary Figure S6 confirm the existence of bimodality, the intrinsic nature of the phenomena and also confirms our original concerns on the convergence of some of the individual ABC simulations published in 2010 (17). It is important to note the similarity between the distributions obtained from the four extended sequences (Figure 5) and distributions shown in the previous section using the ensemble of trajectories (Figures 2 and 3). This implies that convergence issues can be solved using either an ensemble of trajectories representing microsecond conformational sampling, or a single very long simulation. More evidence on these aspects is also provided in the next section.

Additional support for our hypothesis that bimodality is intrinsic, and not only a consequence of the failure of the NN model, comes from the analysis of the experimental naked-DNA structures. Unfortunately, experimental data at the tetramer level are far too scarce as to test bimodality in all different tetramer environments, but at least for one CG tetramer environment (GCGA) clear evidence exists in the naked-DNA structural database supporting intrinsic polymorphism at CpG steps (Supplementary Figure S7). In this work the analysis was conducted up to the tetra-nucleotide level, beyond the current mainstream analysis of DNA. From one side, the bimodality in CpG tetramers appears to be intrinsic, while from the other, the scarcity of data prevents the finding of higher order effects (exa, octa-nucleotides, etc.); thus leaving some uncertainties derived from the use of di- or tetra- oligonucleotide units as transferable blocks for the study of DNA.

### Convergence and the timescale of transitions

**Figure 6.**
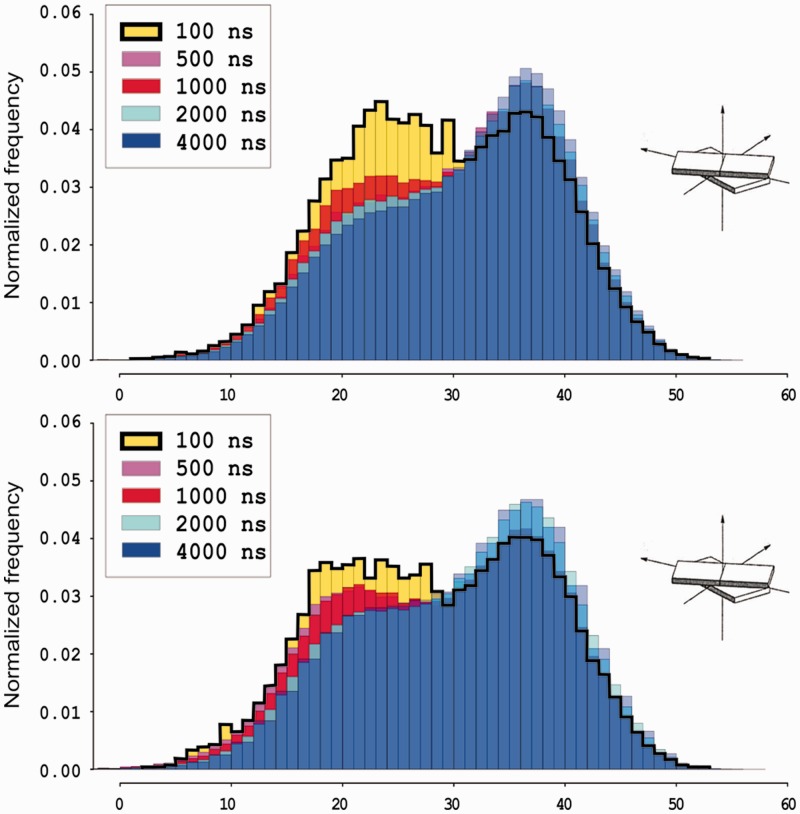
Helical twist for CG dinucleotide steps at bp 3 (top) and bp 9 (bottom) during the 4 µs long simulation of the Drew–Dickerson dodecamer for increasing simulation periods. All analysed steps corresponds to CG steps in the GCGA environment, based on symmetry considerations, bp steps 3 and 9 should be identical.

**Figure 7.**
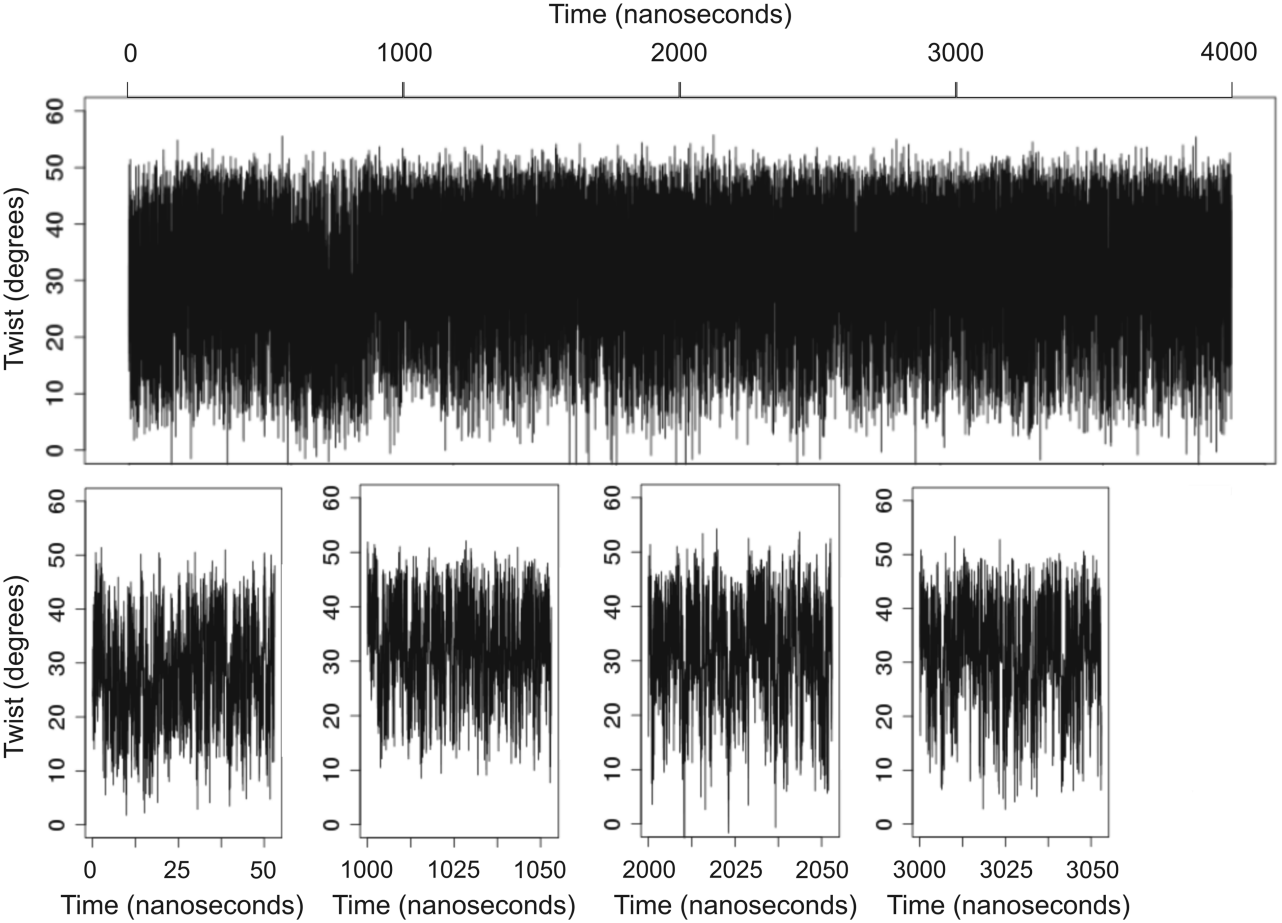
Time evolution of the twist parameter for CG dinucleotide step at bp 3 in the MD simulation of the Drew–Dickerson dodecamer. Changes in twist over the entire simulation (4 µs) are shown in the upper graphic. Four segments of 50 ns taken at *t* = 0 and then every 1000 ns are depicted in the graphics below. Note the transitions in twist between a low and high state. Identical results are obtained with CpG step in bp 9 (data not shown).

The significant changes found between 50 and 750 ns trajectories of the same oligomer (see above) raise questions on the time-dependence of transitions between bimodal states. To answer these questions we performed a very long (4 µs) simulation of Drew–Dickerson dodecamer (see ‘Materials and Methods’ section). The ensemble results are summarized in Figure 6, whereas a detailed time-analysis is displayed in Figure 7. Both plots confirm that bimodality is not an equilibration artefact, given that high-twist/low-twist transitions persist even after 4 µs of trajectory. Analysis of the averages collected for different trajectory lengths show that a single 100 ns trajectory is not converged, since the population of the low-twist state is significantly overestimated. However, distributions seem converged after 500 ns (Figure 6), i.e. within a timescale readily accessible for current simulations. The transitions between high- and low-twist conformations can be considered fast (with average residence time around 0.1 ns) and are coupled to BI↔BII transitions (17). It is important to note (Figure 7) that the frequency of these transitions (5.5 transitions/ns) is constant along the entire trajectory, increasing confidence in the true convergence of MD results presented here.

### Refinement of the elastic model

Once a helical distribution has been characterized as bimodal, the elastic model can be modified, by defining two stiffness matrices associated to each of the states using the BIC fittings to define the reference state of each of the collected snapshots. Accordingly, for a given deformation in a dinucleotide pair step, we have two potential deformation energies, which transform the standard elastic equation (2) into equation (10):
(10)
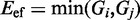

where, 
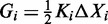
 and 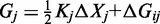.

Here *i* and *j* refer to the two distributions, *K_i,j_* stands for the corresponding stiffness matrices, *ΔX_i,j_* refers to the difference between the given helical parameter and the means of *i* and *j* distributions, and *ΔG_ij_* is the difference in free energy between the peaks of distributions *i* and *j*. Note that if bimodality affects more than one parameter (in a given step) equation (10) needs to be expanded since the number of stiffness matrices and equilibrium values scales with 2*^n^*^−^^1^ (*n* being equal to the number of helical coordinates affected by bimodality).

Although this model can be used for simple energy calculations, it is inappropriate for optimization or dynamics because of the discontinuous derivatives at the crossover point between the two quadratic curves. An elegant solution to this issue was developed by de Marco and Varnai to derive structure-based empirical potential for DNA (36), but here we use a simpler solution based on a continuous function *H_ij_* of the type used for dealing with surface crossing in two-state quantum systems (37), and already exploited for modelling transitions between two conformations using elastic networks (38,39):
(11)
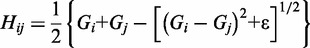

where *ε* is a small positive value.

As a proof of concept, we used this model to evaluate the impact of bimodality in a mesoscopic representation of the flexibility of a CpG step. We found that the stiffness constant matrices for the 6 inter-base pair parameters are relatively insensitive to the changing twist states with the possible exception of the shift-rise coupling constant (Supplementary Figure S8). That is, when going from low to high twist the differences in the pure stiffness constants (roll–roll, slide–slide, etc.) are very low, meaning mild effects in these parameters when changing twist states, except for the shift–shift and rise–rise terms (see the diagonal constants in the bottom matrix of Supplementary Figure S8). The same coupling was also observed when changing slide states in ApG and CpC steps (Supplementary Figure S9). This naturally simplifies the problem of deriving an appropriate bimodal elastic model for DNA. Our calculations reveal that, while for qualitative, and sometimes semi-quantitative, purposes unimodal results can be informative, detailed modelling requires taking bimodality into account. Note that the location of the energy minimum in a unimodal model of a true bimodal distribution is, in reality, a local maximum. Supplementary Figure S10 shows the error can reach 1 kcal/mol at this position. Similarly, the energy penalty assumed in a unimodal model is too small, especially for high-twist values, leading to a significant (and asymmetric) overestimation of the flexibility of DNA.

Although the results in Supplementary Figure S10 point out the improvements achieved by the bimodal model in a test case, we further highlight its utility by calculating the elastic deformation energy connected to intercalation or protein–DNA interactions for specific experimental cases (Supplementary Figure S11). We computed the deformation energy for the family of intercalators used to build Figure 5, for the cases when those intercalators induced in DNA extreme-twist values. The same was done with the few protein–DNA complexes that directly interact with CpG steps producing low-twist values. Our calculations, based on the average distortion energy, reveal that using only the unimodal approach can lead to errors that span from a half to two k_b_T. Unfortunately, to date, there are not many available experimental complexes inducing large deformation in DNA, so data are too scarce to explore these issues by now in more detail.

Considering its simplicity, we believe that the bimodality correction outlined here could easily be introduced into a second generation of elastic models that would be better equipped to reproduce the response of DNA to a wider range of deformations.

## CONCLUSIONS

The combined analysis of X-ray and MD databases provides clear evidence of the existence of binormality in many dinucleotide pair steps, and robust evidence of more intriguing bimodality in the distribution of a small numbers of helical parameters at given dinucleotide pair steps. This result is particularly clear in the case of d(CpG) steps, where two-state polymorphism is strongly supported by both experimental and theoretical evidence. Bimodality is an intrinsic property of some dinucleotide steps (in certain tetranucleotide environments), and not simply due to averaging over non-NN effects. Very long MD trajectories reveal that, even though caution is needed when dealing with individual short trajectories (<100 ns), convergence can be fully achieved in accessible simulation times (around 500 ns), or by using ensembles of trajectories, since bimodal transitions occur on the picosecond to nanosecond timescale. As we have shown, correcting the elastic model to account for this kind of structural polymorphisms is possible; it does not dramatically increase the complexity of the parameterization process and significantly improves the model, especially in the case of extreme deformations.

The analysis of a large set of DNA–protein complexes allows proposing, at least at the bps level, the prevalence of the ‘conformational selection’ paradigm to explain the distortions, out of the DNA canonical values, produced upon protein binding. Examination of the experimental structures lying outside the MD conformation distributions also reveals clear, but infrequent, cases of ‘induced fit’ mechanisms. Nucleosomes, TATA-box binding proteins, some endonucleases, the Catabolite Protein Activator, and some zinc finger proteins are found, almost exclusively, outside the naked-DNA MD conformation distributions, suggesting that the ‘induced fit’ mechanism can be operating in these few cases.

## SUPPLEMENTARY DATA

Supplementary Data are available at NAR Online: Supplementary Tables 1–4 and Supplementary Figures 1–11.

## FUNDING

Spanish Ministry of Science and Innovation [BIO2009-10964 and Consolider E-Science]; European Research Council; Fundación Marcelino Botín; PEDECIBA-Uruguay and SNI (ANII) (to P.D.D.); EMBO fellowship [ALTF 1107 to A.P.]; CNRS (to R.L.). Funding for open access charge: Spanish Ministry of Science and Innovation [BIO2009-10964 and Consolider E-Science]; European Research Council and the Fundación Marcelino Botín.

*Conflict of interest statement*. None declared.

## Supplementary Material

Supplementary Data
