# Supplementary material for: Exploring polymorphisms in B-DNA helical conformations
Source: Nucleic Acids Res. 2012 Sep 24;40(21):10668–78. doi: 10.1093/nar/gks884 (PMC3510489; doi:10.1093/nar/gks884)
Supplement: Supplementary Data [file supp_40_21_10668__index.html]

Exploring polymorphisms in B-DNA helical conformations — Exploring polymorphisms in B-DNA helical conformations — Supplementary Data 

# Exploring polymorphisms in B-DNA helical conformations

## Supplementary Data

files

**Files in this Data Supplement:**

- Supplementary Data - pdf file
