# Supplementary material for: Exploring polymorphisms in B-DNA helical conformations
Source: Nucleic Acids Res. 2012 Sep 24;40(21):10668–78. doi: 10.1093/nar/gks884 (PMC3510489; doi:10.1093/nar/gks884)
Supplement: Supplementary Data [file supp_gks884_nar-00838-f-2012-File003.pdf]

Supplementary Material

EXPLORING POLYMORPHISMS IN B-DNA HELICAL CONFORMATIONS

Pablo D. Dans[1,2], Alberto Pérez[1,3,4], Ignacio Faustino[1], Richard Lavery[5] and Modesto Orozco[1,4,6,*]

[1] Joint IRB-BSC Program on Computational Biology, Institute for Research in Biomedicine, Parc Cientific de Barcelona, Josep Samitier 1-5, Barcelona 08028, Spain.
[2] Institut Pasteur de Montevideo, Mataojo 2020, Montevideo 11400, Uruguay.
[3] Laufer Center for Physical and Quantitative Biology, Stony Brook University, Stony Brook, NY 11794-5252, USA.
[4] Barcelona Supercomputing Centre, Jordi Girona 31, Edifici Torre Girona. Barcelona 08034, Spain.
[5] Bioinformatics: Structures and Interactions, Bases Moléculaires et Structurales des Systèmes Infectieux, Univ. Lyon I/CNRS UMR 5086, IBCP, 7 Passage du Vercors, Lyon 69367, France.
[6] Departament de Bioquimica, Facultat de Biologia, Avgda. Diagonal 647, Barcelona 08028, Spain.

*To whom correspondence should be addressed: Tel: +34 93 403 7155; Fax: +34 93 403 7157; Email: modesto@mmb.pcb.ub.es

Table S1. PDB entries of the X-ray files used in this work organized in five main categories.

Naked-DNA
111D, 112D, 113D, 114D, 122D, 123D, 126D, 145D, 150D, 158D, 167D, 178D, 183D, 194D, 1BD1, 1BNA, 1CGC, 1D23, 1D27, 1D29, 1D49, 1D56, 1D57, 1D62, 1D65, 1D77, 1D80, 1D81, 1D89, 1D8G, 1D97, 1D98, 1D99, 1DA2, 1DA3, 1DCV, 1DN4, 1DN5, 1DN9, 1DNM, 1DOU, 1EDR, 1EHV, 1G8U, 1G8V, 1HQ7, 1ILC, 1JUC, 1M6G, 1N4E, 1QC1, 1SM5, 1T4I, 1ZF5, 1ZFC, 1ZFE, 1ZFF, 1ZFG, 1ZFM, 218D, 233D, 237D, 239D, 242D, 250D, 251D, 252D, 265D, 266D, 270D, 272D, 285D, 286D, 287D, 290D, 291D, 297D, 2P8D, 2QEF, 2QEG, 307D, 351D, 355D, 388D, 389D, 399D, 3DNB, 3F8O, 400D, 423D, 428D, 431D, 436D, 455D, 456D, 457D, 467D, 477D, 4BNA, 4DNB, 5DNB, 7BNA, 9BNA
Enzymes-DNA complexes
1A31, 1A73, 1A74, 1AZ0, 1B94, 1B95, 1B96, 1B97, 1BGB, 1BHM, 1BPX, 1BPY, 1BSS, 1BSU, 1BUA, 1CEZ, 1CKQ, 1CL8, 1CRX, 1CW0, 1CYQ, 1CZ0, 1D02, 1D1U, 1D2I, 1DC1, 1DFM, 1DIZ, 1DMU, 1DNK, 1EBM, 1EMH, 1EMJ, 1EO3, 1EO4, 1EON, 1EOO, 1ERI, 1ESG, 1EWN, 1EYU, 1F0O, 1F44, 1F4R, 1F6O, 1FIU, 1FJX, 1G9Y, 1G9Z, 1HCR, 1HU0, 1I3J, 1I6J, 1IAW, 1IJW, 1IPP, 1JJ6, 1JKO, 1JKR, 1JX4, 1JXL, 1K3W, 1K3X, 1K4T, 1K82, 1KBU, 1L1T, 1L1Z, 1L2B, 1L2C, 1L3S, 1L3T, 1L3U, 1L3V, 1L5U, 1LV5, 1LWV, 1LWW, 1LWY, 1M0E, 1M3H, 1M3Q, 1M5R, 1M5X, 1MA7, 1MOW, 1MUH, 1MUR, 1MUS, 1N39, 1N3A, 1N3E, 1N3F, 1N48, 1N56, 1NJW, 1NJX, 1NJY, 1NJZ, 1NK0, 1NK4, 1NK5, 1NK6, 1NK7, 1NK8, 1NK9, 1NKB, 1NKC, 1NKE, 1NNJ, 1OE4, 1OE5, 1ORN, 1ORP, 1OUP, 1PJI, 1PJJ, 1PM5, 1PT3, 1PVP, 1Q3F, 1QAI, 1QAJ, 1QPS, 1QRH, 1QSS, 1QSY, 1QTM, 1QUM, 1R2Y, 1R2Z, 1R7M, 1RH6, 1RRJ, 1RRQ, 1RRS, 1RV5, 1RVA, 1RVB, 1RVC, 1RXW, 1RYR, 1RYS, 1RZT, 1S0O, 1S10, 1S97, 1S9F, 1SA3, 1SKM, 1SKR, 1SKS, 1SKW, 1SL1, 1SL2, 1SSP, 1STX, 1SUZ, 1SX5, 1SX8, 1SXP, 1SXQ, 1T2T, 1T3N, 1T7P, 1T9I, 1T9J, 1TC3, 1TDZ, 1TEZ, 1TK0, 1TK5, 1TK8, 1TKD, 1TV9, 1TX3, 1U0C, 1U45, 1U47, 1U49, 1U4B, 1UA0, 1UA1, 1V15, 1VRL, 1WTE, 1X9M, 1X9W, 1XC8, 1XC9, 1XHV, 1XO0, 1XSL, 1XSN, 1XSP, 1Y6F, 1Y8Z, 1YA6, 1YF3, 1YQK, 1YQM, 1YQR, 1ZET, 1ZJM, 1ZNS, 1ZTT, 1ZTW, 2AGQ, 2ALZ, 2AOQ, 2AOR, 2AQ4, 2ASD, 2ASJ, 2B0D, 2B0E, 2B9S, 2BAM, 2BCQ, 2BCR, 2BCS, 2BCU, 2BCV, 2BDP, 2BQ3, 2BQR, 2BQU, 2BR0, 2C28, 2C7O, 2C7P, 2C7Q, 2C7R, 2CRX, 2DDG, 2DEM, 2DNJ, 2DP6, 2DPI, 2DPJ, 2DTU, 2E52, 2EA0, 2EX5, 2EZV, 2F5N, 2F5O, 2F5P, 2F5Q, 2FJV, 2FJW, 2FJX, 2FKC, 2FL3, 2FLD, 2FLN, 2FLP, 2FMP, 2FMQ, 2FMS, 2FQZ, 2FVP, 2FVR, 2G1P, 2GB7, 2GE5, 2GIG, 2GIH, 2GII, 2GIJ, 2GWS, 2H7G, 2HHQ, 2HHS, 2HHT, 2HHU, 2HHV, 2HHW, 2HHX, 2HOF, 2HR1, 2HVH, 2HVI, 2HW3, 2I3P, 2I3Q, 2I9G, 2IA6, 2IBK, 2IBS, 2IBT, 2IH2, 2IH4, 2IHM, 2IMW, 2IS6, 2ISO, 2ISP, 2J6S, 2J6U, 2JEF, 2JEG, 2JEI, 2JEJ, 2KTQ, 2NOB, 2NOE, 2NOF, 2NOH, 2NOZ, 2NP6, 2NP7, 2NQ9, 2NQJ, 2O6M, 2OAA, 2ODI, 2OFI, 2OG0, 2OPF, 2OWO, 2OXM, 2OXV, 2OYT, 2P0J, 2P66, 2PFN, 2PFO, 2PFP, 2PFQ, 2PI4, 2PVI, 2PXI, 2PYJ, 2PYL, 2Q10, 2QOJ, 2R2R, 2R2T, 2R8H, 2R8I, 2R9L, 2RDJ, 2SSP, 2UVV, 2UVW, 2UYC, 2UZ4, 2VBJ, 2VBL, 2VBN, 2VBO, 2VE9, 2VLA, 2VOA, 2VS7, 2VS8, 3BAM, 3BDP, 3BEP, 3BI3, 3BIE, 3BJY, 3BKZ, 3BM3, 3BTX, 3BTY, 3BU0, 3C0W, 3C0X, 3C25, 3C2K, 3C2M, 3C58, 3C5F, 3C5G, 3CQ8, 3CRX, 3CVS, 3CVU, 3CWS, 3CWT, 3DPG, 3DVO, 3DW9, 3E3Y, 3E40, 3E54, 3ECP, 3EPG, 3KTQ, 3PVI, 4BDP, 4CRX, 4KTQ, 6MHT, 9MHT
Structural binding proteins-DNA complexes
1AZP, 1AZQ, 1B3T, 1BF4, 1BNZ, 1C8C, 1CA5, 1CA6, 1E3M, 1EQZ, 1EWQ, 1F4K, 1HLV, 1IGN, 1IHF, 1J1V, 1J3E, 1J75, 1JEY, 1KX3, 1KX5, 1M18, 1M19, 1OH6, 1OUZ, 1OWF, 1OWG, 1P3I, 1P3L, 1P51, 1P71, 1P78, 1QBJ, 1S32, 1SFU, 1W0T, 1W0U, 1W7A, 1WB9, 1WBB, 1WBD, 1WD0, 1WD1, 1WTO, 1WTP, 1WTQ, 1WTR, 1WTV, 1WTW, 1WTX, 1XYI, 2CV5, 2EFW, 2HEO, 2HT0, 2I06, 2IIE, 2NL8, 2NQB, 2NTC, 2PYO, 2QHB, 2Z3X, 2ZKD, 2ZO1, 3C1B, 3CLZ, 3F21, 3F8I, 3F8J, 3FDE
Transcription factors-DNA complexes
1A1F, 1A1H, 1A1I, 1A1J, 1A1K, 1A1L, 1A3Q, 1A6Y, 1AAY, 1AIS, 1AKH, 1AM9, 1AU7, 1AWC, 1AZP, 1B72, 1B8I, 1BC7, 1BC8, 1BDT, 1BG1, 1BL0, 1BY4, 1CDW, 1CKT, 1D3U, 1DP7, 1DSZ, 1DU0, 1DUX, 1E3O, 1EGW, 1F2I, 1FJL, 1FYL, 1FYM, 1G2D, 1G2F, 1GA5, 1GD2, 1GTW, 1GU4, 1GU5, 1GXP, 1H6F, 1H89, 1H8A, 1HCQ, 1HWT, 1IG7, 1J59, 1JE8, 1JFT, 1JGG, 1JJ4, 1JK1, 1JK2, 1JNM, 1K61, 1K78, 1K79, 1KU7, 1L3L, 1LAT, 1LE8, 1LLI, 1LLM, 1LMB, 1LQ1, 1MEY, 1MJ2, 1MJM, 1MJO, 1MJQ, 1MNM, 1MNN, 1N6J, 1NFK, 1NH2, 1NKP, 1NLW, 1NVP, 1OZJ, 1P47, 1PDN, 1PER, 1PP7, 1PUE, 1PUF, 1QN3, 1QN4, 1QN5, 1QN6, 1QN7, 1QN8, 1QN9, 1QNA, 1QNB, 1QNC, 1QNE, 1QPZ, 1QRV, 1R0O, 1R4O, 1R71, 1R8E, 1RIO, 1RM1, 1RPE, 1SKN, 1TSR, 1TUP, 1U8B, 1UBD, 1XBR, 1YO5, 1YRN, 1YTF, 1ZAA, 1ZG1, 1ZG5, 1ZME, 1ZRF, 1ZS4, 2A07, 2A66, 2AC0, 2ADY, 2AHI, 2ATA, 2BNW, 2BOP, 2C6Y, 2C7A, 2C9L, 2CGP, 2D5V, 2DGC, 2E1C, 2E42, 2E43, 2ETW, 2EUV, 2EUW, 2EUX, 2EUZ, 2EVF, 2EVG, 2EVH, 2EVI, 2EVJ, 2GEQ, 2H1K, 2H27, 2H7H, 2HAN, 2HAP, 2HDD, 2HOS, 2HOT, 2I13, 2IRF, 2ISZ, 2NLL, 2O49, 2O4A, 2OR1, 2PI0, 2QL2, 2R1J, 2RAM, 2RBF, 2VY1, 2VY2, 3BPY, 3BRD, 3BRF, 3BRG, 3BS1, 3C2I, 3CBB, 3CO6, 3COA, 3COQ, 3CRO, 3E6C, 3ERE, 3EXJ, 3EXL, 3HDD, 3HTS, 6PAX, 9ANT
Small intercalators-DNA complexes
110D, 151D, 152D, 154D, 182D, 198D, 1AGL, 1C9Z, 1D10, 1D11, 1D12, 1D14, 1D15, 1D32, 1D33, 1D35, 1D36, 1D37, 1D38, 1D54, 1D58, 1D67, 1DA0, 1DA9, 1DL8, 1IMR, 1IMS, 1JO2, 1K9G, 1KCI, 1M69, 1NAB, 1P20, 1PFE, 1R68, 1VS2, 1XVK, 1XVN, 1XVR, 1Z3F, 215D, 224D, 234D, 235D, 236D, 245D, 258D, 276D, 277D, 278D, 288D, 2ADW, 2D34, 2DES, 2GB9, 2GJB, 308D, 366D, 367D, 380D, 381D, 385D, 386D, 3C2J, 3GSK, 452D, 454D, 465D, 482D

Table S2. DNA sequences, simulation conditions and origin of the independent Molecular Dynamics trajectories that define the theoretical conformational space.

Sequence	Replicas	Water model	Ions	Simulated time	Origin
**Dodecamers**					
CGCGAAAACGCG	1	TIP3P	Na+ (neutrality)	50 ns	MMB [a]
CGCGAAACCGCG	1	TIP3P	Na+ (neutrality)	50 ns	MMB
CGCGAAAGCGCG	1	TIP3P	Na+ (neutrality)	50 ns	MMB
CGCGAAATCGCG	1	TIP3P	Na+ (neutrality)	50 ns	MMB
CGCGAACACGCG	1	TIP3P	Na+ (neutrality)	50 ns	MMB
CGCGAACCCGCG	1	TIP3P	Na+ (neutrality)	50 ns	MMB
CGCGAACGCGCG	1	TIP3P	Na+ (neutrality)	50 ns	MMB
CGCGAACTCGCG	1	TIP3P	Na+ (neutrality)	50 ns	MMB
CGCGAAGACGCG	1	TIP3P	Na+ (neutrality)	50 ns	MMB
CGCGAAGCCGCG	1	TIP3P	Na+ (neutrality)	50 ns	MMB
CGCGAAGGCGCG	1	TIP3P	Na+ (neutrality)	50 ns	MMB
CGCGAAGTCGCG	1	TIP3P	Na+ (neutrality)	50 ns	MMB
CGCGAATACGCG	1	TIP3P	Na+ (neutrality)	50 ns	MMB
CGCGAATCCGCG	1	TIP3P	Na+ (neutrality)	50 ns	MMB
CGCGAATGCGCG	1	TIP3P	Na+ (neutrality)	50 ns	MMB
CGCGAATTCGCG	1	TIP3P	Na+ (neutrality)	50 ns	MMB
CGCGACAACGCG	1	TIP3P	Na+ (neutrality)	50 ns	MMB
CGCGACACCGCG	1	TIP3P	Na+ (neutrality)	50 ns	MMB
CGCGACAGCGCG	1	TIP3P	Na+ (neutrality)	50 ns	MMB
CGCGACATCGCG	1	TIP3P	Na+ (neutrality)	50 ns	MMB
CGCGACCACGCG	1	TIP3P	Na+ (neutrality)	50 ns	MMB
CGCGACCCCGCG	1	TIP3P	Na+ (neutrality)	50 ns	MMB
CGCGACCGCGCG	1	TIP3P	Na+ (neutrality)	50 ns	MMB
CGCGACCTCGCG	1	TIP3P	Na+ (neutrality)	50 ns	MMB
CGCGACGACGCG	1	TIP3P	Na+ (neutrality)	50 ns	MMB
CGCGACGCCGCG	1	TIP3P	Na+ (neutrality)	50 ns	MMB
CGCGACGGCGCG	1	TIP3P	Na+ (neutrality)	50 ns	MMB
CGCGACGTCGCG	1	TIP3P	Na+ (neutrality)	50 ns	MMB
CGCGAGAACGCG	1	TIP3P	Na+ (neutrality)	50 ns	MMB
CGCGAGACCGCG	1	TIP3P	Na+ (neutrality)	50 ns	MMB
CGCGAGAGCGCG	1	TIP3P	Na+ (neutrality)	50 ns	MMB
CGCGAGATCGCG	1	TIP3P	Na+ (neutrality)	50 ns	MMB
CGCGAGCACGCG	1	TIP3P	Na+ (neutrality)	50 ns	MMB
CGCGAGCCCGCG	1	TIP3P	Na+ (neutrality)	50 ns	MMB
CGCGAGCGCGCG	1	TIP3P	Na+ (neutrality)	50 ns	MMB
CGCGAGCTCGCG	1	TIP3P	Na+ (neutrality)	50 ns	MMB
CGCGATAACGCG	1	TIP3P	Na+ (neutrality)	50 ns	MMB
CGCGATACCGCG	1	TIP3P	Na+ (neutrality)	50 ns	MMB
CGCGATAGCGCG	1	TIP3P	Na+ (neutrality)	50 ns	MMB
CGCGATATCGCG	1	TIP3P	Na+ (neutrality)	50 ns	MMB

CGCGCAAACGCG	1	TIP3P	Na+ (neutrality)	50 ns	MMB
CGCGCAACCGCG	1	TIP3P	Na+ (neutrality)	50 ns	MMB
CGCGCAAGCGCG	1	TIP3P	Na+ (neutrality)	50 ns	MMB
CGCGCAATCGCG	1	TIP3P	Na+ (neutrality)	50 ns	MMB
CGCGCACACGCG	1	TIP3P	Na+ (neutrality)	50 ns	MMB
CGCGCACCCGCG	1	TIP3P	Na+ (neutrality)	50 ns	MMB
CGCGCACGCGCG	1	TIP3P	Na+ (neutrality)	50 ns	MMB
CGCGCACTCGCG	1	TIP3P	Na+ (neutrality)	50 ns	MMB
CGCGCAGACGCG	1	TIP3P	Na+ (neutrality)	50 ns	MMB
CGCGCAGCCGCG	1	TIP3P	Na+ (neutrality)	50 ns	MMB
CGCGCAGGCGCG	1	TIP3P	Na+ (neutrality)	50 ns	MMB
CGCGCAGTCGCG	1	TIP3P	Na+ (neutrality)	50 ns	MMB
CGCGCATACGCG	1	TIP3P	Na+ (neutrality)	50 ns	MMB
CGCGCATGCGCG	1	TIP3P	Na+ (neutrality)	50 ns	MMB
CGCGCCAACGCG	1	TIP3P	Na+ (neutrality)	50 ns	MMB
CGCGCCACCGCG	1	TIP3P	Na+ (neutrality)	50 ns	MMB
CGCGCCAGCGCG	1	TIP3P	Na+ (neutrality)	50 ns	MMB
CGCGCCATCGCG	1	TIP3P	Na+ (neutrality)	50 ns	MMB
CGCGCCCACGCG	1	TIP3P	Na+ (neutrality)	50 ns	MMB
CGCGCCCCCGCG	1	TIP3P	Na+ (neutrality)	50 ns	MMB
CGCGCCCGCGCG	1	TIP3P	Na+ (neutrality)	50 ns	MMB
CGCGCCCTCGCG	1	TIP3P	Na+ (neutrality)	50 ns	MMB
CGCGCCGACGCG	1	TIP3P	Na+ (neutrality)	50 ns	MMB
CGCGCCGGCGCG	1	TIP3P	Na+ (neutrality)	50 ns	MMB
CGCGCGAACGCG	1	TIP3P	Na+ (neutrality)	50 ns	MMB
CGCGCGACCGCG	1	TIP3P	Na+ (neutrality)	50 ns	MMB
CGCGCGAGCGCG	1	TIP3P	Na+ (neutrality)	50 ns	MMB
CGCGCGATCGCG	1	TIP3P	Na+ (neutrality)	50 ns	MMB
CGCGCGCACGCG	1	TIP3P	Na+ (neutrality)	50 ns	MMB
CGCGCGCGCGCG	1	TIP3P	Na+ (neutrality)	50 ns	MMB
CGCGCTAACGCG	1	TIP3P	Na+ (neutrality)	50 ns	MMB
CGCGCTAGCGCG	1	TIP3P	Na+ (neutrality)	50 ns	MMB
CGCGGAAACGCG	1	TIP3P	Na+ (neutrality)	50 ns	MMB
CGCGGAACCGCG	1	TIP3P	Na+ (neutrality)	50 ns	MMB
CGCGGAAGCGCG	1	TIP3P	Na+ (neutrality)	50 ns	MMB
CGCGGAATCGCG	1	TIP3P	Na+ (neutrality)	50 ns	MMB
CGCGGACACGCG	1	TIP3P	Na+ (neutrality)	50 ns	MMB
CGCGGACCCGCG	1	TIP3P	Na+ (neutrality)	50 ns	MMB
CGCGGACGCGCG	1	TIP3P	Na+ (neutrality)	50 ns	MMB
CGCGGACTCGCG	1	TIP3P	Na+ (neutrality)	50 ns	MMB
CGCGGAGACGCG	1	TIP3P	Na+ (neutrality)	50 ns	MMB
CGCGGAGCCGCG	1	TIP3P	Na+ (neutrality)	50 ns	MMB
CGCGGAGGCGCG	1	TIP3P	Na+ (neutrality)	50 ns	MMB
CGCGGATACGCG	1	TIP3P	Na+ (neutrality)	50 ns	MMB
CGCGGATGCGCG	1	TIP3P	Na+ (neutrality)	50 ns	MMB

**Tetrakaidecamers**					
CGGACGACCGCGCG	2	TIP3P	Na+ (neutrality)	100 ns	MMB
CGGCCGAACGCGCG	2	TIP3P	Na+ (neutrality)	100 ns	MMB
CGGACGGGCGAGCG	2	TIP3P	Na+ (neutrality)	100 ns	MMB
CGGCCGGACGTGCG	2	TIP3P	Na+ (neutrality)	100 ns	MMB
CGGGCGCTCGAGCG	2	TIP3P	Na+ (neutrality)	100 ns	MMB
**Octakaidecamers**					
GCCTATAAACGCCTATAA	1	TIP3P	Na+ (neutrality)	100 ns	MMB
CTAGGTGGATGACTCATT	1	TIP3P	Na+ (neutrality)	100 ns	MMB
CACGGAACCGGTTCCGTG	1	TIP3P	Na+ (neutrality)	100 ns	MMB
GGCGCGCACCACGCGCGG	1	TIP3P	Na+ (neutrality)	100 ns	MMB
GCAAAAAAAAAAAAAAGC	1	SPC/E	K+ (0.15 M)	50-100 ns	ABC [D]
GCACAAACAAACAAACGC	1	SPC/E	K+ (0.15 M)	50-100 ns	ABC
GCATAAATAAATAAATGC	1	SPC/E	K+ (0.15 M)	50-100 ns	ABC
GCAGAGAGAGAGAGAGGC	1	SPC/E	K+ (0.15 M)	50-100 ns	ABC
GCCGAGCGAGCGAGCGGC	1	SPC/E	K+ (0.15 M)	50-100 ns	ABC
GCCTAGCTAGCTAGCTGC	1	SPC/E	K+ (0.15 M)	50-100 ns	ABC
GCGAAGGAAGGAAGGAGC	1	SPC/E	K+ (0.15 M)	50-100 ns	ABC
GCGCAGGCAGGCAGGCGC	1	SPC/E	K+ (0.15 M)	50-100 ns	ABC
GCGTAGGTAGGTAGGTGC	1	SPC/E	K+ (0.15 M)	50-100 ns	ABC
GCTCAGTCAGTCAGTCGC	1	SPC/E	K+ (0.15 M)	50-100 ns	ABC
GCTGAGTGAGTGAGTGGC	1	SPC/E	K+ (0.15 M)	50-100 ns	ABC
GCGCATGCATGCATGCGC	1	SPC/E	K+ (0.15 M)	50-100 ns	ABC
GCAGCAAGCAAGCAAGGC	1	SPC/E	K+ (0.15 M)	50-100 ns	ABC
GCATCAATCAATCAATGC	1	SPC/E	K+ (0.15 M)	50-100 ns	ABC
GCCGCGCGCGCGCGCGGC	1	SPC/E	K+ (0.15 M)	50-100 ns	ABC
GCGACGGACGGACGGAGC	1	SPC/E	K+ (0.15 M)	50-100 ns	ABC
GCGCCGGCCGGCCGGCGC	1	SPC/E	K+ (0.15 M)	50-100 ns	ABC
GCGTCGGTCGGTCGGTGC	1	SPC/E	K+ (0.15 M)	50-100 ns	ABC
GCTACGTACGTACGTAGC	1	SPC/E	K+ (0.15 M)	50-100 ns	ABC
GCTGCGTGCGTGCGTGGC	1	SPC/E	K+ (0.15 M)	50-100 ns	ABC
GCAAGAAAGAAAGAAAGC	1	SPC/E	K+ (0.15 M)	50-100 ns	ABC
GCACGAACGAACGAACGC	1	SPC/E	K+ (0.15 M)	50-100 ns	ABC
GCACGAACGAACGAACGC	15	SPC/E	Na+ (0.06 M)	50 ns	MMB
GCACGAACGAACGAACGC	15	SPC/E	Na+ (0.50 M)	50 ns	MMB
GCATGAATGAATGAATGC	1	SPC/E	K+ (0.15 M)	50-100 ns	ABC
GCTAGATAGATAGATAGC	1	SPC/E	K+ (0.15 M)	50-100 ns	ABC
GCGAGGGAGGGAGGGAGC	1	SPC/E	K+ (0.15 M)	50-100 ns	ABC
GCGCGGGCGGGCGGGCGC	1	SPC/E	K+ (0.15 M)	50-100 ns	ABC
GCGGGGGGGGGGGGGGGC	1	SPC/E	K+ (0.15 M)	50-100 ns	ABC
GCGTGGGTGGGTGGGTGC	1	SPC/E	K+ (0.15 M)	50-100 ns	ABC
GCACTAACTAACTAACGC	1	SPC/E	K+ (0.15 M)	50-100 ns	ABC
GCAGTAAGTAAGTAAGGC	1	SPC/E	K+ (0.15 M)	50-100 ns	ABC
GCATTAATTAATTAATGC	1	SPC/E	K+ (0.15 M)	50-100 ns	ABC
GCTATATATATATATAGC	1	SPC/E	K+ (0.15 M)	50-100 ns	ABC

GCGATCGATCGATCGAGC	1	SPC/E	K+ (0.15 M)	50-100 ns	ABC
GCGATGGATGGATGGAGC	1	SPC/E	K+ (0.15 M)	50-100 ns	ABC
GCGCTGGCTGGCTGGCGC	1	SPC/E	K+ (0.15 M)	50-100 ns	ABC
GCGTTGGTTGGTTGGTGC	1	SPC/E	K+ (0.15 M)	50-100 ns	ABC
GCTATGTATGTATGTAGC	1	SPC/E	K+ (0.15 M)	50-100 ns	ABC
GCTCTGTCTGTCTGTCGC	1	SPC/E	K+ (0.15 M)	50-100 ns	ABC
GCTGTGTGTGTGTGTGGC	1	SPC/E	K+ (0.15 M)	50-100 ns	ABC

[a] Trajectories retrieved from our local database. [b] Trajectories coming from the ABC consortium.

Table S3. Amount of data (N), average values, and standard deviation of the six intra-strand base pair parameters for the 10 unique bps.[a]

Step	N	Twist	Tilt	Roll	Shift	Slide	Rise
	60	35.6 ± 4.3	-1.4 ± 2.7	1.0 ± 3.8	0.0 ± 0.3	-0.2 ± 0.4	3.3 ± 0.1
AA	*850*	*33.4 ± 5.9*	*-1.2 ± 3.0*	*2.5 ± 7.1*	*0.1 ± 0.5*	*0.0 ± 0.6*	*3.3 ± 0.2*
	**1 M**[b]	**35.4 ± 5.4**	**-2.6 ± 4.1**	**0.3 ± 5.6**	**-0.3 ± 0.6**	**-0.3 ± 0.6**	**3.3 ± 0.3**
	12	35.6 ± 2.3	-0.5 ± 2.2	0.4 ± 3.6	-0.1 ± 0.6	-0.2 ± 0.3	3.3 ± 0.1
AC	*670*	*31.5 ± 3.9*	*0.7 ± 3.4*	*2.0 ± 4.1*	*0.2 ± 0.6*	*-0.6 ± 0.4*	*3.2 ± 0.2*
	**1 M**	**32.0 ± 5.1**	**-0.7 ± 4.0**	**-0.6 ± 5.9**	**0.1 ± 0.7**	**-0.6 ± 0.5**	**3.3 ± 0.3**
	17	29.7 ± 5.9	-0.4 ± 4.0	5.7 ± 4.9	0.4 ± 0.4	0.3 ± 0.8	3.4 ± 0.3
AG	*700*	*32.4 ± 4.7*	*-1.1 ± 3.6*	*3.2 ± 5.7*	*0.1 ± 0.7*	*-0.2 ± 0.3*	*3.3 ± 0.3*
	**1 M**	**33.5 ± 6.0**	**-2.5 ± 4.2**	**3.1 ± 5.9**	**-0.4 ± 0.7**	**-0.6 ± 0.7**	**3.4 ± 0.3**
	62	32.0 ± 3.6	0.3 ± 2.2	-1.2 ± 3.1	0.1 ± 0.4	-0.6 ± 0.3	3.3 ± 0.2
AT	*890*	*30.0 ± 4.7*	*0.2 ± 2.9*	*1.6 ± 5.3*	*0.0 ± 0.5*	*-0.6 ± 0.4*	*3.2 ± 0.3*
	**1 M**	**30.4 ± 4.2**	**0.0 ± 3.8**	**-0.5 ± 5.1**	**0.0 ± 0.7**	**-0.8 ± 0.4**	**3.3 ± 0.3**
	30	41.9 ± 8.7	0.7 ± 2.7	0.7 ± 7.0	0.1 ± 0.2	1.6 ± 1.0	3.3 ± 0.2
CA	*730*	*35.9 ± 5.5*	*0.3 ± 3.9*	*4.6 ± 6.8*	*-0.1 ± 0.6*	*0.6 ± 1.0*	*3.4 ± 0.3*
	**1 M**	**29.6 ± 8.3**	**0.2 ± 4.8**	**10.3 ± 6.3**	**-0.2 ± 0.7**	**-0.2 ± 0.6**	**3.1 ± 0.4**
	36	33.8 ± 5.6	1.9 ± 3.4	4.3 ± 4.6	0.0 ± 0.5	0.7 ± 1.0	3.4 ± 0.3
CC	*560*	*33.7 ± 4.6*	*0.9 ± 4.7*	*4.3 ± 5.5*	*0.1 ± 0.7*	*-0.2 ± 0.8*	*3.4 ± 0.3*
	**1 M**	**32.7 ± 6.6**	**0.0 ± 4.4**	**4.9 ± 5.7**	**0.2 ± 0.7**	**-0.7 ± 0.9**	**3.5 ± 0.3**
	205	33.0 ± 5.1	0.3 ± 4.5	3.4 ± 7.7	0.1 ± 0.5	0.5 ± 0.6	3.3 ± 0.3
CG	*860*	*32.5 ± 5.7*	*-0.2 ± 4.2*	*4.6 ± 5.9*	*0.0 ± 0.7*	*0.4 ± 0.8*	*3.3 ± 0.3*
	**1 M**	**29.8 ± 8.8**	**0.0 ± 5.2**	**9.3 ± 6.0**	**0.0 ± 0.7**	**0.0 ± 0.5**	**3.1 ± 0.4**
	35	38.9 ± 3.9	-0.8 ± 3.3	2.3 ± 3.8	0.0 ± 0.4	0.0 ± 0.5	3.4 ± 0.2
GA	*805*	*35.4 ± 4.7*	*-1.4 ± 3.6*	*2.3 ± 5.5*	*-0.2 ± 0.6*	*-0.1 ± 0.6*	*3.3 ± 0.2*
	**1 M**	**36.6 ± 6.2**	**-1.6 ± 4.5**	**1.6 ± 5.9**	**-0.4 ± 0.7**	**-0.3 ± 0.7**	**3.4 ± 0.3**
	125	39.7 ± 4.4	-0.5 ± 3.7	-5.2 ± 6.0	-0.2 ± 0.8	0.3 ± 0.4	3.5 ± 0.2
GC	*940*	*35.0 ± 5.2*	*-0.2 ± 3.7*	*0.6 ± 5.7*	*0.0 ± 0.7*	*-0.1 ± 0.6*	*3.3 ± 0.3*
	**1 M**	**35.7 ± 5.1**	**0.0 ± 4.0**	**-1.3 ± 5.8**	**0.0 ± 0.7**	**-0.4 ± 0.6**	**3.5 ± 0.3**
	19	41.7 ± 6.3	-0.1 ± 3.5	1.4 ± 5.0	-0.2 ± 0.4	0.7 ± 0.8	3.4 ± 0.2
TA	*680*	*35.7 ± 7.5*	*0.1 ± 3.6*	*3.2 ± 7.2*	*0.0 ± 0.6*	*0.3 ± 1.0*	*3.4 ± 0.4*
	**1 M**	**28.9 ± 8.4**	**0.0 ± 5.1**	**10.0 ± 7.4**	**0.0 ± 0.9**	**-0.2 ± 0.8**	**3.2 ± 0.4**

[a] The naked-DNA structures, all the PDB structures (italics), and results from MD simulations with parmbsc0 (bold) are shown. Values for simulations were obtained from time averages computed for individual steps in each sequence. Rotational parameters are in degrees (Twist, Tilt, Roll), and translational ones (Shift, Slide, Rise) in Å. Complementary steps (e.g. AG and CT) have the same average except for a change in sign of Shift and Tilt, and the same standard deviation. [b] One million configurations were taken from the trajectories for each bps (see Methods).

Table S4. Average values, standard deviations, and mixture proportion of the normal components obtained with BIC.[a]

Step	Twist	Tilt	Roll	Shift	Slide	Rise
AA	*avg_1 = 26.0 ± 7.6* [a]<br>*avg_2 = 35.4 ± 3.3*<br>*p_r=0.21*<br>**avg_1 = 32.4 ± 5.2**<br>**avg_2 = 38.6 ± 3.4**<br>**p_r=0.52**	*avg_1 = -2.1 ± 3.7*<br>*avg_2 = -0.6 ± 2.1*<br>*p_r=0.41*<br>**avg_1 = -2.5 ± 4.1**	*avg_1 = 0.1 ± 4.0*<br>*avg_2 = 9.2 ± 9.1*<br>*p_r=0.73*<br>**avg_1 = -0.3 ± 4.6**<br>**avg_2 = 1.0 ± 6.5**<br>**p_r=0.53**	*avg_1 = 0.0 ± 0.6*<br>*avg_2 = 0.1 ± 0.3*<br>*p_r=0.36*<br>**avg_1 = -0.7 ± 0.5**<br>**avg_2 = 0.0 ± 0.5**<br>**p_r=0.48**	*avg_1 = -0.2 ± 0.3*<br>*avg_2 = 0.3 ± 0.6*<br>*p_r=0.61*<br>**avg_1 = -0.7 ± 0.5**<br>**avg_2 = 0.1 ± 0.4**<br>**p_r=0.50**	*avg_1 = 3.2 ± 0.1*<br>*avg_2 = 3.3 ± 0.3*<br>*p_r=0.54*<br>**avg_1 = 3.2 ± 0.2**<br>**avg_2 = 3.4 ± 0.3**<br>**p_r=0.53**
AC	*avg_1 = 32 ± 4*<br>**avg_1 = 30.0 ± 5.7**<br>**avg_2 = 33.6 ± 3.7**<br>**p_r=0.46**	*avg_1 = 0.4 ± 4.7*<br>*avg_2 = 0.9 ± 2.5*<br>*p_r=0.34*<br>**avg_1 = -0.7 ± 4.0**	*avg_1 = 1.9 ± 3.3*<br>*avg_2 = 2.1 ± 6.7*<br>*p_r=0.84*<br>**avg_1 = -1.7 ± 6.5**<br>**avg_2 = 0.5 ± 4.9**<br>**p_r=0.47**	*avg_1 = 0.0 ± 0.6*<br>*avg_2 = 0.6 ± 0.3*<br>*p_r=0.64*<br>**avg_1 = -0.4 ± 0.6**<br>**avg_2 = 0.6 ± 0.5**<br>**p_r=0.50**	*avg_1 = -0.6 ± 0.3*<br>*avg_2 = -0.5 ± 0.6*<br>*p_r=0.64*<br>**avg_1 = -0.7 ± 0.6**<br>**avg_2 = -0.5 ± 0.4**<br>**p_r=0.46**	*avg_1 = 3.2 ± 0.2*<br>*avg_2 = 3.6 ± 0.4*<br>*p_r=0.94*<br>**avg_1 = 3.3 ± 0.3**<br>**avg_2 = 3.3 ± 0.4**<br>**p_r=0.52**
AG	*avg_1 = 31.5 ± 5.9*<br>*avg_2 = 33.2 ± 3.1*<br>*p_r=0.46*<br>**avg_1 = 30.2 ± 5.6**<br>**avg_2 = 37.4 ± 3.5**<br>**p_r=0.54**	*avg_1 = -1.1 ± 5.1*<br>*avg_2 = -1.0 ± 2.6*<br>*p_r=0.34*<br>**avg_1 = -2.5 ± 4.2**	*avg_1 = 0.5 ± 8.6*<br>*avg_2 = 4.2 ± 3.7*<br>*p_r=0.27*<br>**avg_1 = 3.1 ± 5.9**	*avg_1 = -0.1 ± 0.9*<br>*avg_2 = 0.2 ± 0.4*<br>*p_r=0.49*<br>**avg_1 = -0.8 ± 0.6**<br>**avg_2 = 0.1 ± 0.6**<br>**p_r=0.49**	*avg_1 = -0.2 ± 0.8*<br>*avg_2 = -0.2 ± 0.4*<br>*p_r=0.37*<br>**avg_1 = -1.2 ± 0.4**<br>**avg_2 = -0.2 ± 0.5**<br>**p_r=0.48**	*avg_1 = 3.3 ± 0.2*<br>*avg_2 = 3.7 ± 0.5*<br>*p_r=0.93*<br>**avg_1 = 3.3 ± 0.3**<br>**avg_2 = 3.5 ± 0.3**<br>**p_r=0.53**
AT	*avg_1 = 28.6 ± 6.0*<br>*avg_2 = 31.1 ± 2.7*<br>*p_r=0.46*<br>**avg_1 = 28.7 ± 5.4**<br>**avg_2 = 31.3 ± 3.2**<br>**p_r=0.33**	*avg_1 = -0.3 ± 4.5*<br>*avg_2 = 0.3 ± 2.0*<br>*p_r=0.26*<br>**avg_1 = 0.0 ± 3.8**	*avg_1 = 0.6 ± 3.5*<br>*avg_2 = 6.0 ± 8.6*<br>*p_r=0.81*<br>**avg_1 = -1.1 ± 4.1**<br>**avg_2 = 0.1 ± 6.0**<br>**p_r=0.53**	*avg_1 = -0.2 ± 0.6*<br>*avg_2 = 0.1 ± 0.4*<br>*p_r=0.47*<br>**avg_1 = 0.0 ± 0.7**	*avg_1 = -0.6 ± 0.2*<br>*avg_2 = -0.5 ± 0.7*<br>*p_r=0.80*<br>**avg_1 = -0.9 ± 0.5**<br>**avg_2 = -0.7 ± 0.3**<br>**p_r=0.36**	*avg_1 = 3.2 ± 0.2*<br>*avg_2 = 3.3 ± 0.7*<br>*p_r=0.91*<br>**avg_1 = 3.2 ± 0.2**<br>**avg_2 = 3.3 ± 0.3**<br>**p_r=0.56**
CA	*avg_1 = 36 ± 6*<br>**avg_1 = 23.7 ± 6.1**<br>**avg_2 = 34.9 ± 5.5**<br>**p_r=0.46**	*avg_1 = 0 ± 4*<br>**avg_1 = -1.6 ± 4.8**<br>**avg_2 = 1.9 ± 4.2**<br>**p_r=0.48**	*avg_1 = 2.2 ± 9.0*<br>*avg_2 = 6.4 ± 3.5*<br>*p_r=0.43*<br>**avg_1 = 8.3 ± 6.9**<br>**avg_2 = 12.1 ± 5.1**<br>**p_r=0.47**	*avg_1 = -0.1 ± 0.6*<br>**avg_1 = -0.2 ± 0.8**<br>**avg_2 = -0.1 ± 0.6**<br>**p_r=0.48**	*avg_1 = 0.4 ± 0.8*<br>**avg_1 = -0.4 ± 0.5**<br>**avg_2 = 0.0 ± 0.7**<br>**p_r=0.54**	*avg_1 = 3.3 ± 0.2*<br>*avg_2 = 3.6 ± 0.4*<br>*p_r=0.85*<br>**avg_1 = 2.9 ± 0.3**<br>**avg_2 = 3.4 ± 0.4**<br>**p_r=0.48**
CC	*avg_1 = 34 ± 5*<br>**avg_1 = 30.0 ± 5.4**<br>**avg_2 = 37.6 ± 4.1**<br>**p_r=0.57**	*avg_1 = 0.5 ± 3.3*<br>*avg_2 = 3.6 ± 8.8*<br>*p_r=0.87*<br>**avg_1 = 0.0 ± 4.4**	*avg_1 = 2.6 ± 6.9*<br>*avg_2 = 5.7 ± 3.5*<br>*p_r=0.45*<br>**avg_1 = 4.9 ± 5.7**	*avg_1 = 0.1 ± 0.7*<br>**avg_1 = -0.3 ± 0.6**<br>**avg_2 = 0.7 ± 0.6**<br>**p_r=0.53**	*avg_1 = -0.2 ± 0.8*<br>**avg_1 = -1.5 ± 0.4**<br>**avg_2 = -0.2 ± 0.7**<br>**p_r=0.49**	*avg_1 = 3.4 ± 0.2*<br>*avg_2 = 3.4 ± 0.5*<br>*p_r=0.86*<br>**avg_1 = 3.4 ± 0.3**<br>**avg_2 = 3.6 ± 0.3**<br>**p_r=0.52**
CG	avg_1 = 26.0 ± 1.4<br>avg_2 = 34.7 ± 4.2<br>p_r=0.20<br>*avg_1 = 31.2 ± 6.6*<br>*avg_2 = 34.7 ± 2.5*<br>*p_r=0.63*<br>**avg_1 = 23.2 ± 5.9**<br>**avg_2 = 36.4 ± 5.1**<br>**p_r=0.49**	avg_1 = 0.1 ± 3.9<br>*avg_1 = -0.5 ± 9.9*<br>*avg_2 = -0.2 ± 3.4*<br>*p_r=0.07*<br>**avg_1 = 0.0 ± 5.2**	avg_1 = 2.9 ± 6.0<br>*avg_1 = 2.9 ± 6.8*<br>*avg_2 = 6.9 ± 3.3*<br>*p_r=0.57*<br>**avg_1 = 7.1 ± 6.4**<br>**avg_2 = 11.2 ± 4.8**<br>**p_r=0.48**	avg_1 = 0.1 ± 0.5<br>*avg_1 = 0.0 ± 0.7*<br>**avg_1 = 0.0 ± 0.7**	avg_1 = 0.4 ± 1.1<br>avg_2 = 0.5 ± 0.3<br>p_r=0.19<br>*avg_1 = 0.3 ± 1.0*<br>*avg_2 = 0.5 ± 0.4*<br>*p_r=0.42*<br>**avg_1 = -0.2 ± 0.4**<br>**avg_2 = 0.0 ± 0.7**<br>**p_r=0.54**	avg_1 = 3.2 ± 0.2<br>avg_2 = 3.5 ± 0.3<br>p_r=0.62<br>*avg_1 = 3.3 ± 0.2*<br>*avg_2 = 3.8 ± 0.5*<br>*p_r=0.97*<br>**avg_1 = 2.8 ± 0.3**<br>**avg_2 = 3.3 ± 0.4**<br>**p_r=0.49**
GA	*avg_1 = 33.5 ± 5.5*<br>*avg_2 = 37.1 ± 2.9*<br>*p_r=0.47*<br>**avg_1 = 33.3 ± 6.4**<br>**avg_2 = 39.8 ± 3.6**<br>**p_r=0.48**	*avg_1 = -1.5 ± 2.4*<br>*avg_2 = -1.4 ± 5.0*<br>*p_r=0.62*<br>**avg_1 = -1.6 ± 4.4**	*avg_1 = 0.9 ± 7.4*<br>*avg_2 = 3.3 ± 3.1*<br>*p_r=0.43*<br>**avg_1 = 1.6 ± 5.9**	*avg_1 = -0.2 ± 0.3*<br>*avg_2 = -0.2 ± 0.7*<br>*p_r=0.43*<br>**avg_1 = -0.8 ± 0.5**<br>**avg_2 = 0.0 ± 0.6**<br>**p_r=0.52**	*avg_1 = -0.1 ± 0.3*<br>*avg_2 = 0.0 ± 0.9*<br>*p_r=0.62*<br>**avg_1 = -0.8 ± 0.5**<br>**avg_2 = 0.2 ± 0.5**<br>**p_r=0.47**	*avg_1 = 3.3 ± 0.1*<br>*avg_2 = 3.4 ± 0.3*<br>*p_r=0.66*<br>**avg_1 = 3.3 ± 0.3**<br>**avg_2 = 3.4 ± 0.4**<br>**p_r=0.53**
GC	avg_1 = 36.1 ± 5.6<br>avg_2 = 40.7 ± 2.9<br>p_r=0.25<br>*avg_1 = 37 ± 5*<br>**avg_1 = 31.8 ± 6.6**<br>**avg_2 = 36.6 ± 4.1**<br>**p_r=0.18**	avg_1 = -1 ± 4<br>*avg_1 = -0.2 ± 2.5*<br>*avg_2 = -0.2 ± 4.8*<br>*p_r=0.55*<br>**avg_1 = 0.0 ± 4.0**	avg_1 = -6.2 ± 4.5<br>avg_2 = 3.1 ± 7.8<br>p_r=0.88<br>*avg_1 = -1.1 ± 6.5*<br>*avg_2 = 2.9 ± 3.3*<br>*p_r=0.57*<br>**avg_1 = -2.3 ± 6.7**<br>**avg_2 = -0.4 ± 4.7**<br>**p_r=0.47**	avg_1 = -1.0 ± 0.3<br>avg_2 = 0.4 ± 0.4<br>p_r=0.42<br>*avg_1 = -0.5 ± 0.5*<br>*avg_2 = 0.5 ± 0.5*<br>*p_r=0.51*<br>**avg_1 = -0.4 ± 0.5**<br>**avg_2 = 0.4 ± 0.5**<br>**p_r=0.50**	avg_1 = -0.2 ± 0.6<br>avg_2 = 0.5 ± 0.2<br>p_r=0.35<br>*avg_1 = -0.2 ± 0.8*<br>**avg_1 = -0.7 ± 0.6**<br>**avg_2 = -0.2 ± 0.5**<br>**p_r=0.48**	avg_1 = 3.5 ± 0.2<br>*avg_1 = 3.3 ± 0.2*<br>*avg_2 = 3.4 ± 0.6*<br>*p_r=0.94*<br>**avg_1 = 3.5 ± 0.2**<br>**avg_2 = 3.5 ± 0.3**<br>**p_r=0.53**
TA	*avg_1 = 30.2 ± 7.3*<br>*avg_2 = 39.7 ± 4.5*<br>*p_r=0.42*<br>**avg_1 = 23.6 ± 6.7**<br>**avg_2 = 34.0 ± 6.4**<br>**p_r=0.49**	*avg_1 = -0.4 ± 6.2*<br>*avg_2 = 0.2 ± 2.6*<br>*p_r=0.20*<br>**avg_1 = 0.0 ± 5.1**	*avg_1 = 3 ± 7*<br>**avg_1 = 6.5 ± 7.2**<br>**avg_2 = 13.3 ± 5.8**<br>**p_r=0.49**	*avg_1 = 0.0 ± 0.4*<br>*avg_2 = 0.1 ± 0.7*<br>*p_r=0.55*<br>**avg_1 = 0.0 ± 0.9**	*avg_1 = -0.4 ± 0.6*<br>*avg_2 = 1.2 ± 0.6*<br>*p_r=0.61*<br>**avg_1 = -0.7 ± 0.4**<br>**avg_2 = 0.2 ± 0.7**<br>**p_r=0.48**	*avg_1 = 3.3 ± 0.2*<br>*avg_2 = 4.4 ± 0.3*<br>*p_r=0.92*<br>**avg_1 = 3.0 ± 0.3**<br>**avg_2 = 3.4 ± 0.4**<br>**p_r=0.51**

[a] The naked-DNA structures (only shown for CG and GC), all the PDB structures (italics), and results from MD simulations with parmbsc0 (bold) are shown. Rotational parameters are in degrees (Twist, Tilt, Roll), and translational ones (Shift, Slide, Rise) in Å. Complementary steps (e.g. AG and CT) have the same average except for a change in sign of Shift and Tilt, and the same standard deviation.

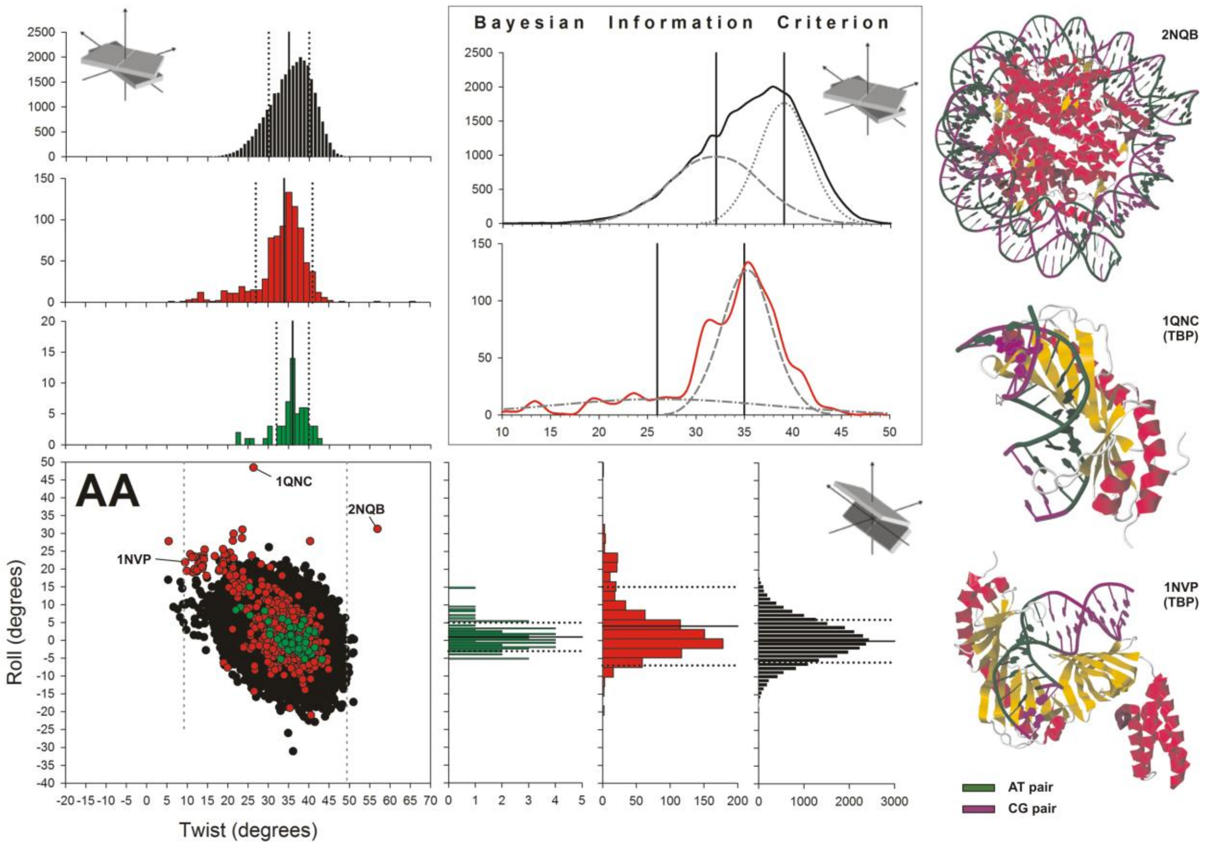

Figure S1. Scatter plot in the twist-roll plane of the AA base pair step for MD simulations (black), all PDB files (red), and naked-DNA structures (green). Some experimental structures outlier's were labeled with the corresponding PDB code and depicted at the right of the graphic. In the scatter plot the gray vertical dashed lines define the range of values used for twist in the BIC analysis. Histograms on the edges of the scatter plot represent the non-normalized distributions (count). The upper right quadrant shows the results of the analysis carried out with BIC for twist. The Gaussian curves in dashed gray are a qualitative representation of the normal components obtained, while the vertical lines represent the corresponding averages (solid) and standard deviations (dashed). For sake of clarity, only a subset of 25000 conformations from the MD simulations was used to build the scatter plot and corresponding histograms.

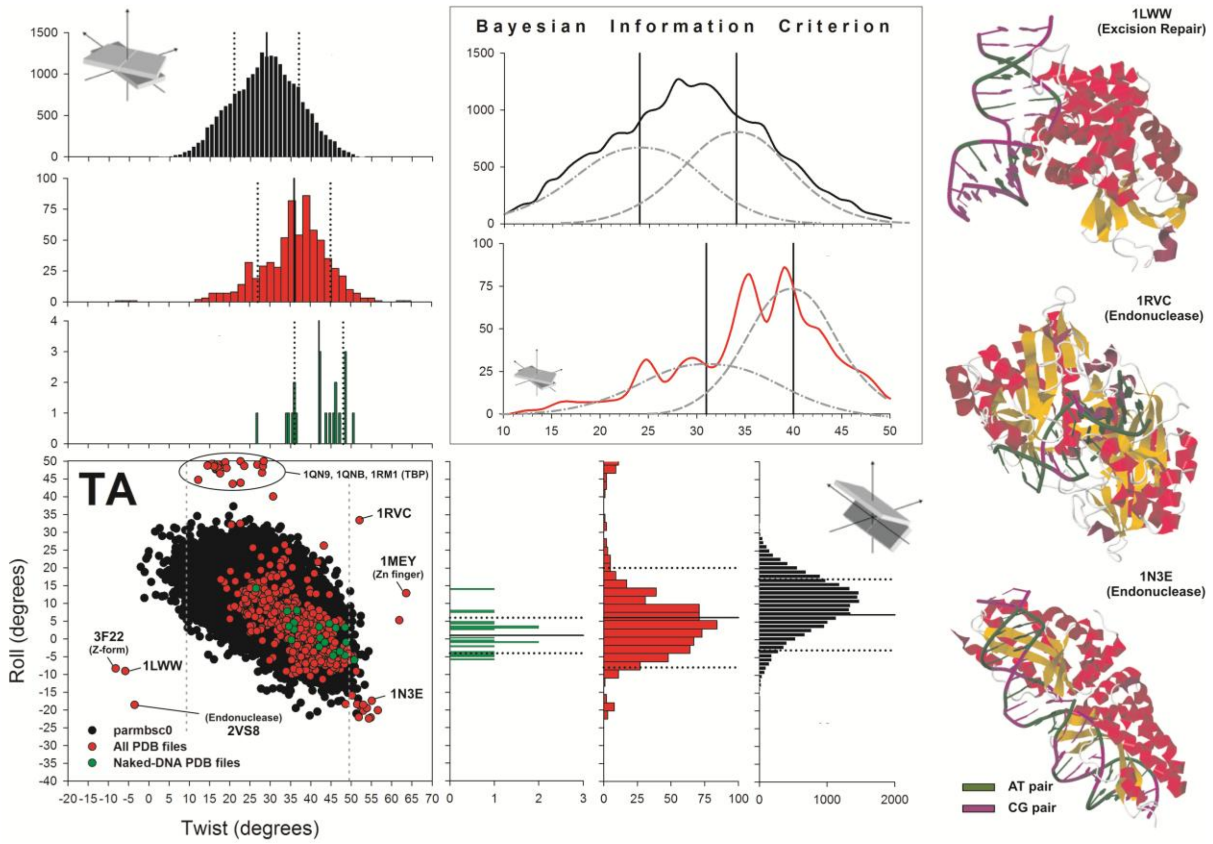

Figure S2. Scatter plot in the twist-roll plane of the TA base pair step for MD simulations (black), all PDB files (red), and naked-DNA structures (green). Some experimental structure outliers were labeled with the corresponding PDB code and depicted at the right of the graphic. In the scatter plot, the gray vertical dashed lines define the range of values used for twist in the BIC analysis. Histograms on the edges of the scatter plot represent the non-normalized distributions (count). The upper right quadrant shows the results of the analysis carried out with BIC for twist. The Gaussian curves in dashed gray are a qualitative representation of the normal components obtained, while the vertical lines represent the corresponding averages (solid) and standard deviations (dashed). For sake of clarity, only a subset of 25000 conformations from the MD simulations was used to build the scatter plot and corresponding histograms.

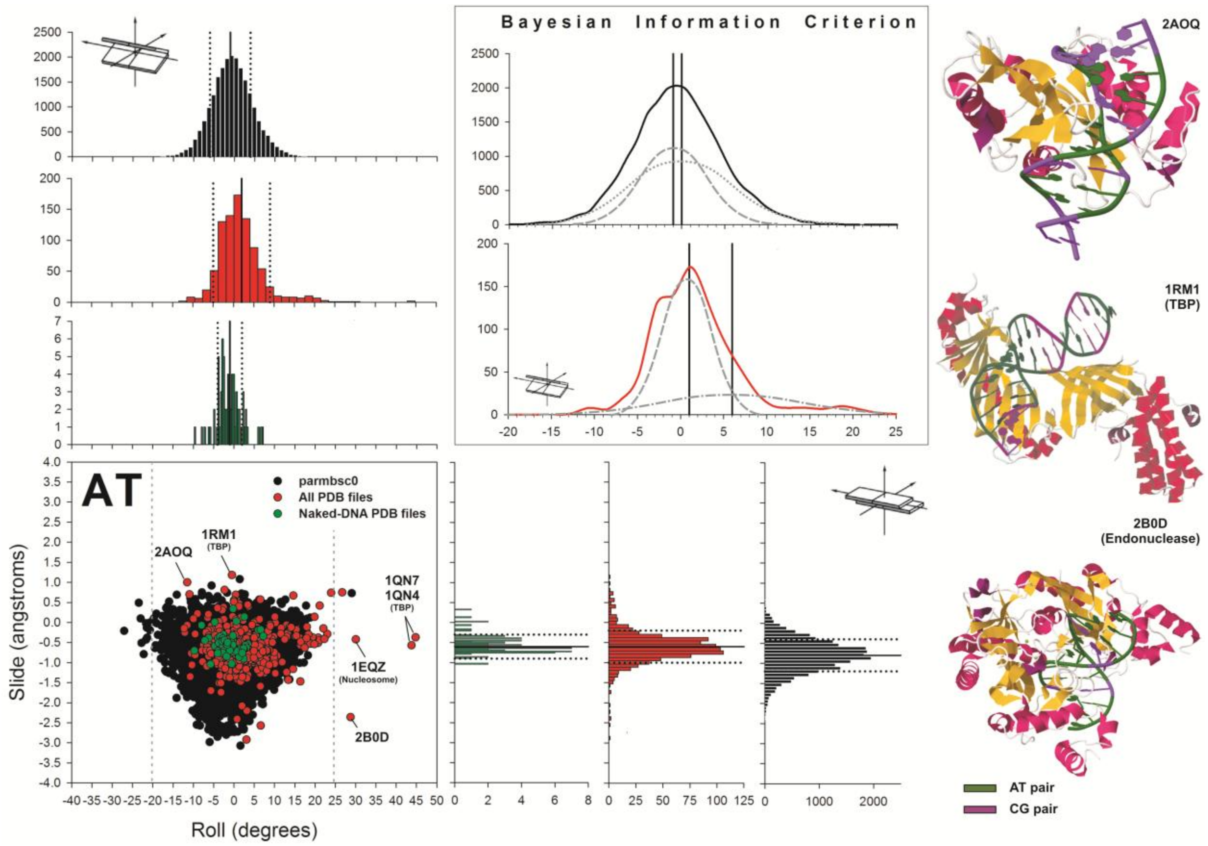

Figure S3. Scatter plot in the roll-slide plane of the AT base pair step for MD simulations (black), all PDB files (red), and naked-DNA structures (green). Some experimental structure outliers were labeled with the corresponding PDB code and depicted at the right of the graphic. In the scatter plot, the gray vertical dashed lines define the range of values used for twist in the BIC analysis. Histograms on the edges of the scatter plot represent the non-normalized distributions (count). The upper right quadrant shows the results of the analysis carried out with BIC for twist. The Gaussian curves in dashed gray are a qualitative representation of the normal components obtained, while the vertical lines represent the corresponding averages (solid) and standard deviations (dashed). For sake of clarity, only a subset of 25000 conformations from the MD simulations was used to build the scatter plot and corresponding histograms.

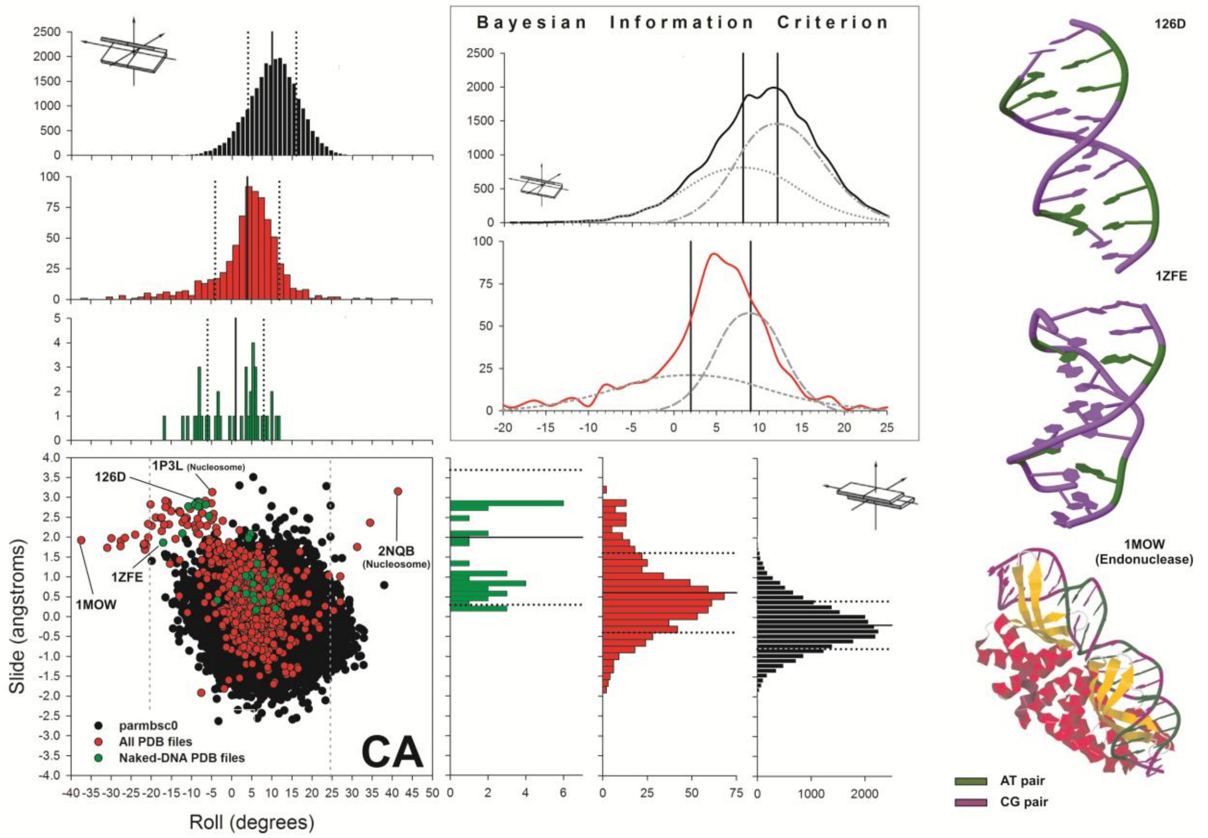

Figure S4. Scatter plot in the roll-slide plane of the CA base pair step for MD simulations (black), all PDB files (red), and naked-DNA structures (green). Some experimental structure outliers were labeled with the corresponding PDB code and depicted at the right of the graphic. In the scatter plot, the gray vertical dashed lines define the range of values used for twist in the BIC analysis. Histograms on the edges of the scatter plot represent the non-normalized distributions (count). The upper right quadrant shows the results of the analysis carried out with BIC for twist. The Gaussian curves in dashed gray are a qualitative representation of the normal components obtained, while the vertical lines represent the corresponding averages (solid) and standard deviations (dashed). For sake of clarity, only a subset of 25000 conformations from the MD simulations was used to build the scatter plot and corresponding histograms.

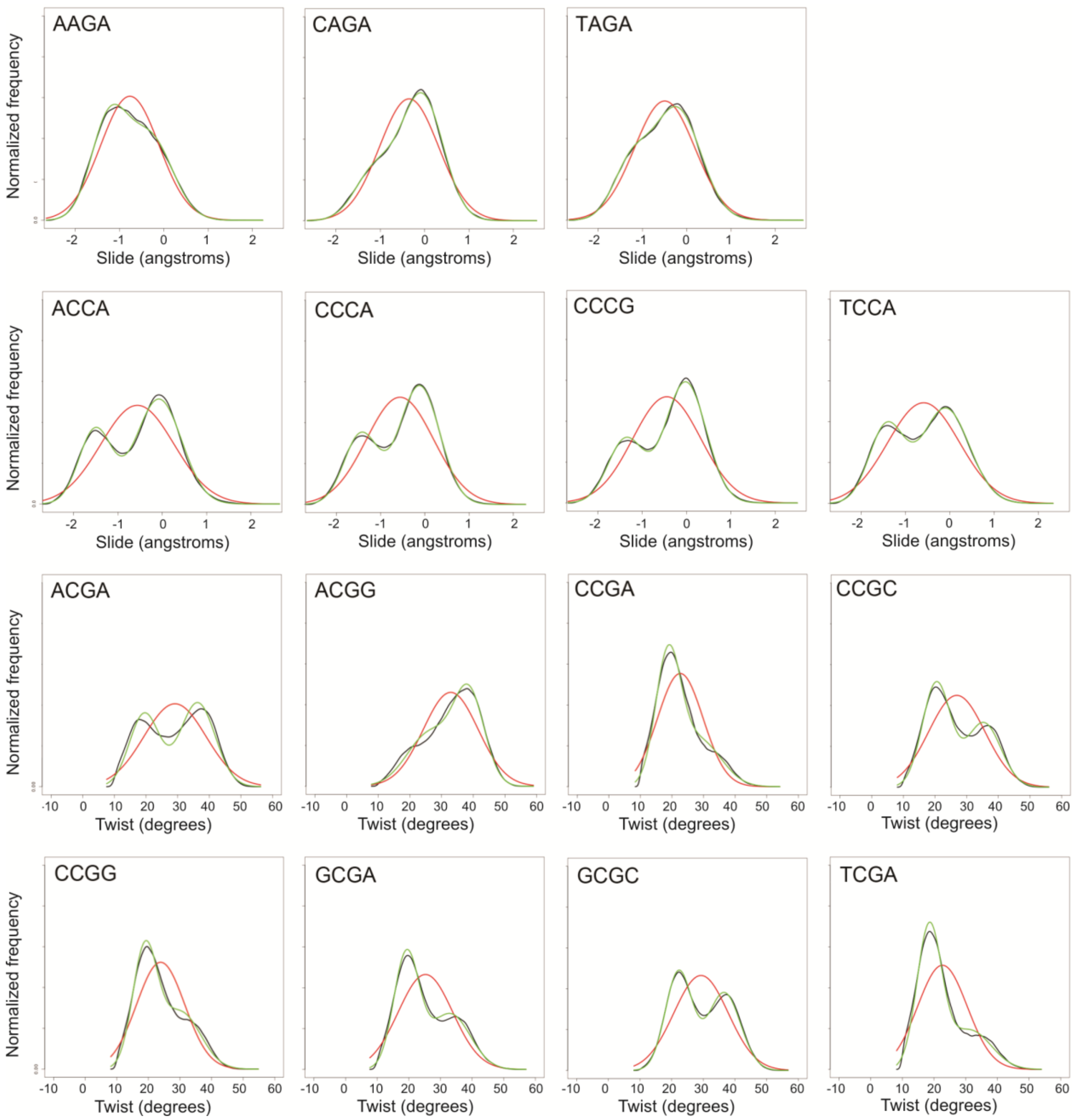

Figure S5. Tetranucleotides where bimodality was found. The tetramers containing the AG bps (first row), the CC bps (second row) and the CG bps (third and fourth rows) are shown. The smoothed distributions are depicted in black, while the BIC results with one and two components (one or two Gaussians) are represented by a red and green line respectively.

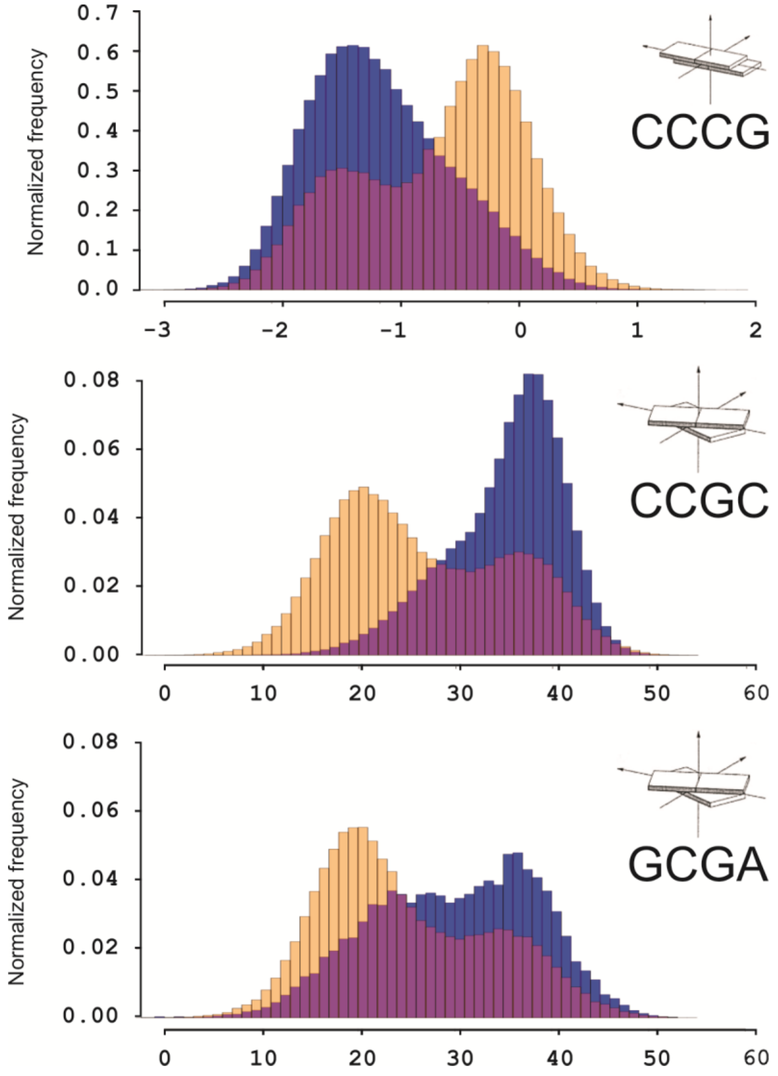

Figure S6. Comparison, at the tetramer level, of the normalized frequency distributions obtained from the original ABC simulations (yellow bars) and the four sequences extended in this work (blue bars). Three tetrads are shown: CCCG for slide, CCGC and GCGA for twist (from top to bottom). Note that the overlap between the two histograms is shown in purple.

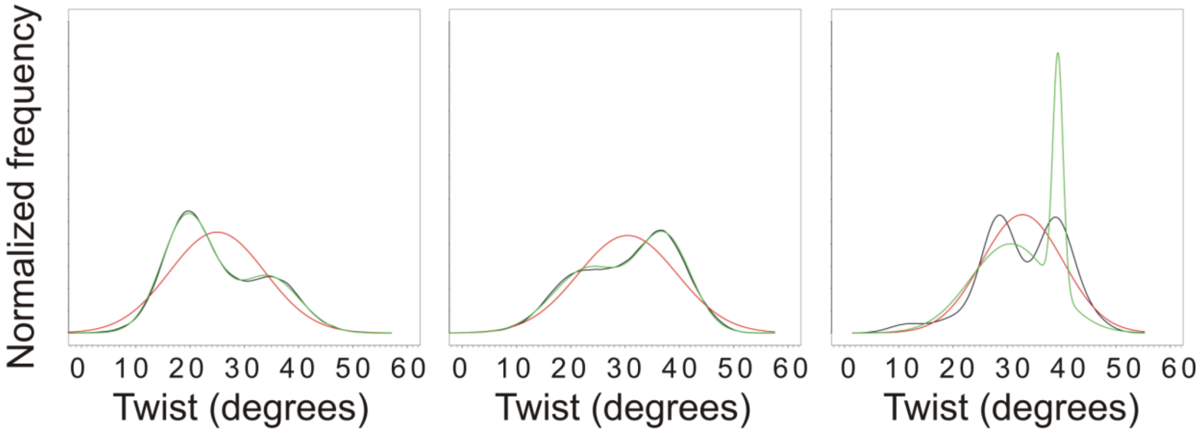

Figure S7. Comparison of the normalized frequency distributions for GCGA tetramer found in the ABC set (left), 4 microsecond Drew-Dickerson dodecamer simulation (center), and the naked-DNA structures (right). The smoothed distributions are shown in black, while the BIC results with one and two components (one or two Gaussians) are represented by a red and green line respectively.

High twist

	shift	slide	rise	tilt	roll	twist
shift	1.20826	0.29399	-0.02602	-0.12542	-0.00843	-0.02388
slide	0.29400	2.03444	0.87443	0.01365	0.00920	-0.08720
rise	-0.02599	0.87446	6.64290	0.04722	-0.05131	-0.19836
tilt	-0.12542	0.01365	0.04723	0.02882	0.00123	0.00063
roll	-0.00843	0.00920	-0.05132	0.00123	0.01413	0.00557
twist	-0.02388	-0.08720	-0.19835	0.00063	0.00557	0.03329
mean	-0.39	0.18	3.32	-3.3	6.2	37.2

Low twist

	shift	slide	rise	tilt	roll	twist
shift	1.32229	0.23206	0.03165	-0.06598	0.00056	0.01538
slide	0.23206	1.96569	0.30034	0.01777	-0.00709	-0.02988
rise	0.03165	0.30036	7.27889	0.05101	-0.10750	-0.21548
tilt	-0.06599	0.01777	0.05101	0.02992	0.00030	0.00003
roll	0.00056	-0.00709	-0.10749	0.00030	0.02055	0.00808
twist	0.01538	-0.02988	-0.21547	0.00003	0.00808	0.02015
mean	-0.19	-0.12	2.96	-1.9	10.4	23.8

High - Low

	shift	slide	rise	tilt	roll	twist
shift	-0.11403	0.06193	-0.05767	-0.05944	-0.00899	-0.03926
slide	0.06194	0.06875	0.57409	-0.00412	0.01629	-0.05732
rise	-0.05764	0.57410	-0.63599	-0.00379	0.05619	0.01712
tilt	-0.05943	-0.00412	-0.00378	-0.00110	0.00093	0.00060
roll	-0.00899	0.01629	0.05617	0.00093	-0.00642	-0.00251
twist	-0.03926	-0.05732	0.01712	0.00060	-0.00251	0.01314

Figure S8. Stiffness matrix in the helical space for the high (top), and low (middle) twist states in CpG steps. Stiffness constants associated with pure rotational (twist, roll and tilt) and translational (rise, shift and slide) deformations are shown in red. The average values of each helical parameter are reported in blue. The bottom matrix represents the difference between the high and low twist stiffness constants. Note that the CpG steps were analyzed going in the 5' to 3' direction in strand I. Using the second strand would change sign for shift and tilt and therefore the coupling terms associated with these parameters would be 0.

AG

High slide

	shift	slide	rise	tilt	roll	twist
shift	1.41372	0.09079	0.54193	-0.12124	0.02474	0.05513
slide	0.09080	2.38362	1.32711	-0.00830	0.00083	-0.10546
rise	0.54197	1.32709	9.50829	-0.28777	0.00498	-0.21059
tilt	-0.12124	-0.00831	-0.28777	0.04785	-0.00301	0.00260
roll	0.02473	0.00083	0.00498	-0.00301	0.01846	0.00884
twist	0.05513	-0.10546	-0.21061	0.00260	0.00884	0.03336
mean	-0.15	-0.26	3.37	-0.1	5.2	35.1

Low slide

	shift	slide	rise	tilt	roll	twist
shift	1.78052	0.05928	0.28733	-0.07094	0.01024	0.02858
slide	0.05928	4.02298	2.12701	-0.01700	-0.04269	-0.19849
rise	0.28733	2.12702	8.65556	-0.26753	-0.02470	-0.20286
tilt	-0.07094	-0.01701	-0.26754	0.04498	-0.00258	0.00030
roll	0.01024	-0.04269	-0.02470	-0.00258	0.02271	0.00845
twist	0.02858	-0.19848	-0.20286	0.00030	0.00845	0.04054
mean	0.15	-1.40	3.53	1.5	6.0	30.8

CC

High slide

	shift	slide	rise	tilt	roll	twist
shift	1.37649	-0.00035	-0.07613	-0.08089	0.02230	0.02250
slide	-0.00036	2.83461	1.84228	0.01308	0.01588	-0.12177
rise	-0.07614	1.84231	8.19154	-0.10394	0.00002	-0.22576
tilt	-0.08089	0.01308	-0.10395	0.04128	-0.00060	0.00106
roll	0.02230	0.01588	0.00003	-0.00060	0.01580	0.00668
twist	0.02250	-0.12177	-0.22576	0.00105	0.00668	0.03208
mean	0.01	-0.47	3.35	-0.2	3.0	34.0

Low slide

	shift	slide	rise	tilt	roll	twist
shift	1.90628	-0.12168	0.04349	-0.04699	-0.01286	0.01957
slide	-0.12167	5.11825	2.22564	-0.00563	-0.01985	-0.21159
rise	0.04349	2.22564	7.35514	-0.16392	-0.03174	-0.22702
tilt	-0.04699	-0.00563	-0.16392	0.04176	-0.00173	-0.00054
roll	-0.01286	-0.01985	-0.03174	-0.00173	0.02210	0.00736
twist	0.01957	-0.21159	-0.22702	-0.00054	0.00736	0.04102
mean	0.06	-1.48	3.52	0.6	5.4	30.1

Figure S9. Stiffness matrix in the helical space for the high and low slide states in ApG (top) and CpC (bottom) steps. Stiffness constants associated with pure rotational (twist, roll and tilt) and translational (rise, shift and slide) deformations are shown in red. The averages values of each helical parameter are reported in blue. Note that both steps were analyzed going in the 5’ to 3’ direction in strand I. Using the second strand would change sign for shift and tilt and therefore the coupling terms associated with these parameters would be 0.

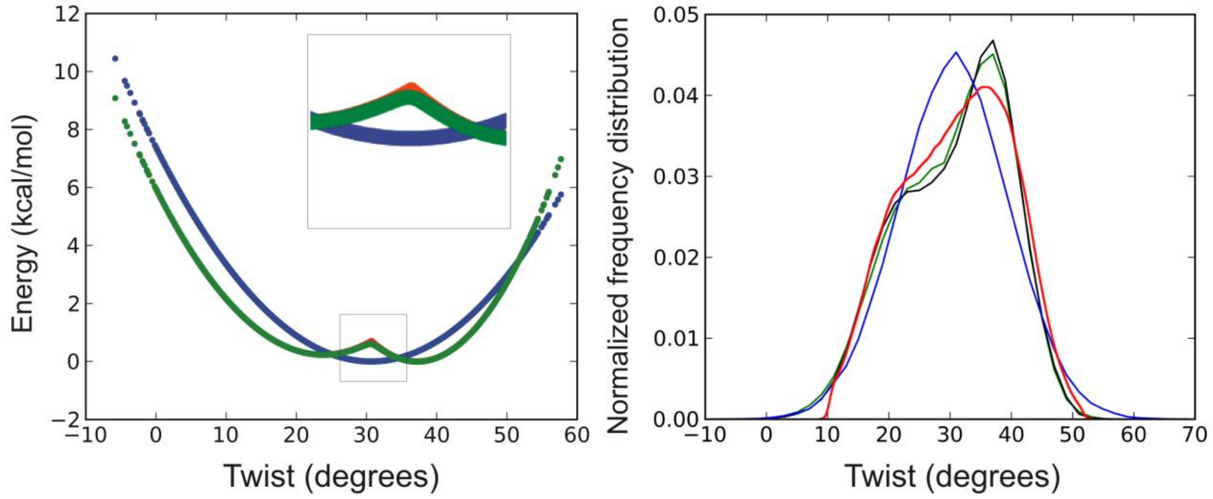

Figure S10. Extending the elastic model to account for bimodality in the CpG steps. Left: the energy profile for the unimodal (blue), the exact bimodal (computed with eq. 10, red), and the continuous bimodal (computed with eq. 11 using ε=0.05, green) mesoscopic models. Right: Normalized frequency distributions for the dataset (black), the unimodal model (blue), the bimodal model (green), and the ensemble of MD simulations for the CpG bps (red).

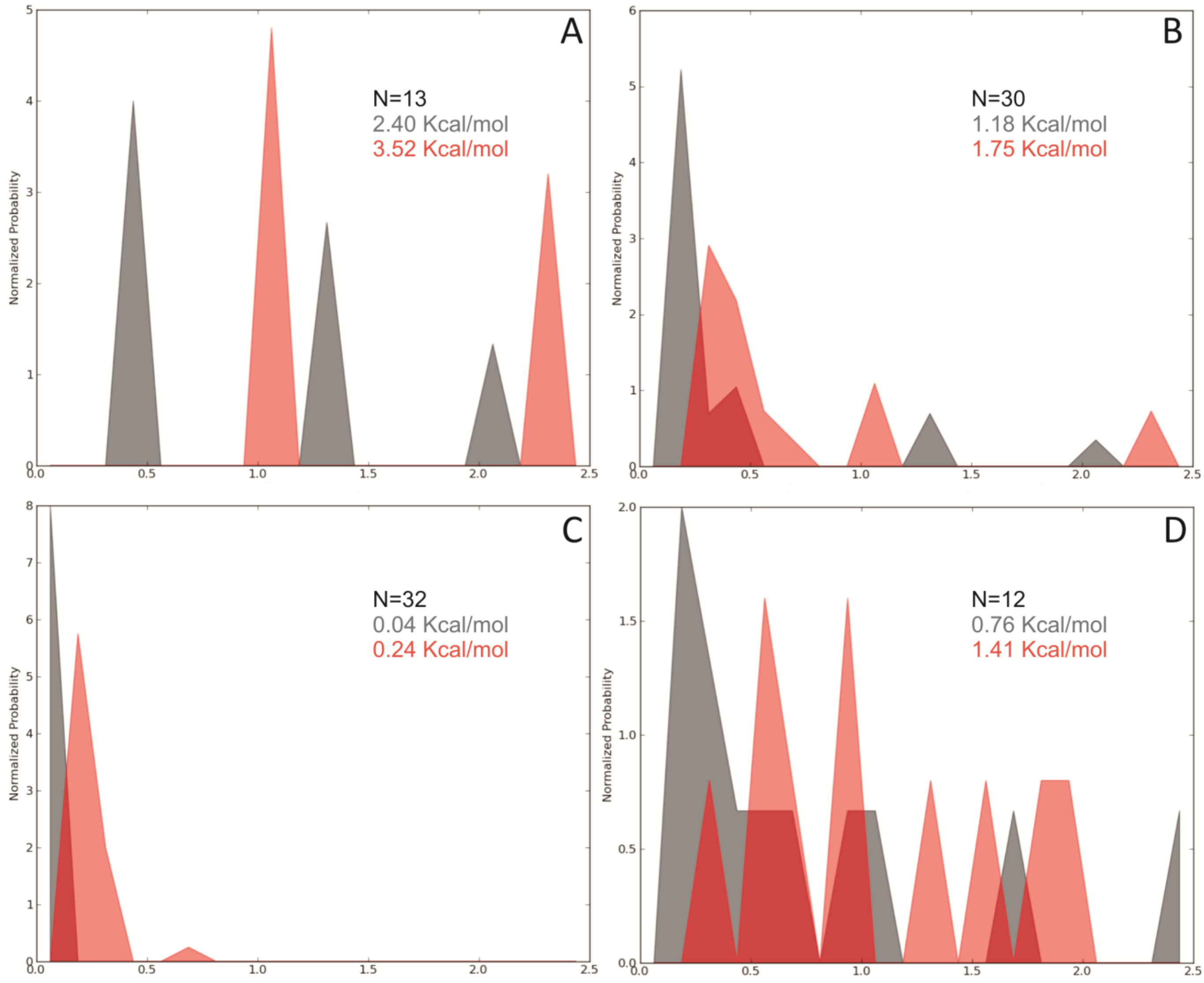

Figure S11. Histograms of deformation energies (Kcal/mol) in experimental CpG steps using the unimodal (red), and bimodal (gray) mesoscopic models. A) Intercalators that induced in DNA very low twist (< 20 degrees); B) DNA-intercalators with twist lower than 25º; C) DNA-intercalators with twist greater than 35º; and D) Protein-DNA complexes that trigger low twist conformations (< 25 degrees). The number of data used to build the histograms is shown (N) together with the corresponding average deformation energies. Note that only ten X-ray structures of protein-DNA complexes interact directly at CpG steps producing an untwisting of the DNA. Those structures belong to two categories: Structural DNA binding proteins (PDB ID: 1AZP, 1BF4, 1WD1, 1WTO, 1WTP, 1WTR, and 1WTW), and transcription factors (PDB ID: 1JFT, 1QPZ, and 1ZRF).
